# Endozoochory by Black Rhinoceroses Enhances Germination of a Key Arid Savanna Tree Species

**DOI:** 10.1002/ece3.71951

**Published:** 2025-09-01

**Authors:** O. E. Jones, H. Beckett, A. J. Abraham, N. P. Makunga, G. F. Midgley

**Affiliations:** ^1^ School for Climate Studies Stellenbosch University Matieland South Africa; ^2^ Centre for Ecological Dynamics in a Novel Biosphere (ECONOVO), Section of Ecoinformatics and Biodiversity, Department of Biology Aarhus University Aarhus C Denmark; ^3^ School of Informatics, Computing, and Cyber Systems Northern Arizona University Flagstaff Arizona USA; ^4^ Department of Botany and Zoology Stellenbosch University Matieland South Africa

**Keywords:** arid savanna, browsing megaherbivore, *Diceros bicornis*, gut passage, Kalahari, recruitment

## Abstract

Megaherbivores are typically regarded as agents of top‐down control, limiting woody encroachment through destructive foraging. Yet they also possess traits and engage in behaviours that facilitate plant success. For example, megaherbivores can act as effective endozoochorous seed dispersers. However, studies on facilitative roles are heavily biased towards the African savanna elephant ( *Loxodonta africana* ), with little attention paid to other species or to effects beyond germination, across early ontogenic stages. The African black rhinoceros ( *Diceros bicornis* ), an obligate browser that exhibits frugivory and defecates in fixed dung middens, may offer ecologically distinct dispersal services. We conducted controlled experiments to test whether black rhino interactions with *Vachellia erioloba* , a leguminous tree of ecological importance in arid savannas, enhance germination, early seedling development or seedling resilience to herbivory. Germination was compared among dung‐derived seeds, untreated controls and chemically scarified seeds. Seedling growth was assessed in dung versus sand and under simulated black rhino herbivory. Dung‐derived seeds germinated most steadily and produced the highest cumulative germination (+40%) over the longest period (+13 days). Growth trials revealed that dung substrates did not enhance initial growth. Rather, seedlings being older conferred greater resilience to biomass loss than exposure to different substrate conditions. Our results provide the first experimental evidence of an apparent mutualism between black rhino and *V. erioloba* . This relationship is not driven by enhanced seedling development through legacy effects of gut passage, nor by dung conditions, as expected. Instead, it stems from gut passage effects on germination. In addition to increasing total germination, gut passage accelerates germination and extends the germination period, producing a seedling cohort with both older individuals and greater age variation—a population structure that may enhance persistence beyond the germination bottleneck. This research supports a more nuanced view of megaherbivores as both disturbance agents and mutualists in arid ecosystems.

## Graphical abstract

This study presents the first experimental evidence of a mutualism between black rhinoceroses and *Vachellia erioloba* , addressing the prevailing bias towards the African savanna elephant in megaherbivore research. We show that gut passage enhances germination timing, duration and cohort structure, producing older, more resilient seedlings—a trait that may promote woody recruitment in arid savannas. These findings broaden our understanding of megaherbivores as both disturbance agents and plant mutualists in dryland ecosystems.

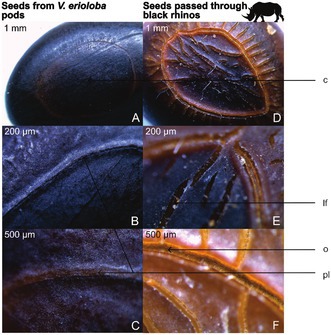

## 1 Introduction

Open ecosystems, such as grasslands and savannas, hold immense ecological, economic and cultural value, and play an essential role in regulating Earth's carbon cycle (Bond 2019; Parr et al. 2014; Stevens et al. 2017). However, these systems face widespread transformation due to anthropogenic pressures. One of the most pervasive threats is woody plant encroachment: the densification of woody vegetation and closure of open habitats (García Criado et al. 2020). Driven primarily by rising atmospheric CO_2_, woody plant encroachment has cascading impacts on biodiversity, productivity and global climate feedbacks (Kristensen et al. 2024; O'Connor et al. 2014; Münch et al. 2019; Smit and Prins 2015). These challenges are pronounced in arid savannas, where vegetation dynamics are highly responsive to disturbance regimes (Case et al. 2019; Scholes 2020).

The extinction (Gill et al. 2009) or functional loss (Ripple et al. 2015) of Pleistocene megaherbivores has likely contributed to the widespread vegetation shifts. Megaherbivores (> 1000 kg) have traditionally been viewed as suppressors of woody plant encroachment, reducing woody proliferation through destructive foraging, trampling and debarking (Hempson et al. 2015; Holdo et al. 2013; Midgley and Bond 2015; Midgley et al. 2020). However, these species are increasingly recognised as ‘ecosystem engineers’ (Enquist et al. 2020; Haynes 2012; Malhi et al. 2016; Owen‐Smith 1987), whose interactions with vegetation are not solely antagonistic. In addition to top‐down control, megaherbivores engage in behaviours that may have facilitative ecological outcomes for woody plants. For example, seed ingestion may enhance woody plant dispersal and germination (Anderson et al. 2014). To build a more nuanced and ecologically realistic understanding of megaherbivore systemic contributions, it is necessary to understand the full spectrum of their interactions with vegetation—including those that may be mutualistic.

Allometric scaling relationships of seed ingestion, gut passage time and daily movement distances suggest that megaherbivores are particularly effective seed facilitators (Abraham et al. 2020; Miller 1995; Pires et al. 2018; Stavi et al. 2015). Compared to smaller species, megaherbivores ingest a greater absolute number and diversity of seeds, which they transport over especially long distances (Bunney et al. 2017; Campos‐Arceiz and Blake 2011; Doughty et al. 2013; Haynes 2012; Hobbs 1996), often with increased survival due to their less destructive dentition (Stavi et al. 2015). Most megaherbivores are hindgut fermenters (with the exception of giraffes), characterised by lower gut acidity and proportionally shorter retention times than ruminants. This digestive strategy exposes seeds to mild mechanical and chemical scarification, enhancing germination potential while preserving viability (Stavi et al. 2015). Indeed, Campos‐Arceiz and Blake (2011) found that germination rates of *Balanites wilsoniana* increased from 3% to 57% after ingestion by African elephants. Moreover, their ability to consume large seeds offers plants an evolutionary avenue to circumvent the trade‐off between seed size and dispersal effectiveness (Doughty et al. 2016; Pires et al. 2018).

Megaherbivores can facilitate plant success across multiple ontogenetic life stages. Their impacts may influence seedlings abilities to overcome both biotic and abiotic bottlenecks to germination, recruitment and early establishment, potentially offering a survival strategy well suited to the unpredictable rainfall and disturbance patterns of arid savannas (February et al. 2019).

Consumption and subsequent dispersal of seeds (endozoochory) plays a central role in influencing when and where seeds germinate, maintaining genetic structure, linking populations and driving species distribution across heterogeneous natural systems (Clark 1998; Zhu et al. 2023). Thus, as climate shifts intensify, long‐distance dispersal agents like megaherbivores may play a crucial role in assisting plant species to track habitable environmental niches (Frei et al. 2018; Fricke et al. 2022). This might be particularly true in semi‐arid environments, where climate shifts are severe and where herbivores disperse seeds further because they need to cover more ground to meet their biological needs, so occupy larger home ranges (Huang et al. 2023).

An advantage of herbivore‐mediated germination is its function as a bet hedging survival strategy (Anderson et al. 2012; Baskin and Baskin 2001): Only a subset of seeds are freed from pods and stimulated to germinate, while the remainder lie dormant in a persistent seed bank (Odirile et al. 2019). This prohibits the loss of an entire generation after germination following temporarily favourable conditions. Endozoochory may also help seeds circumvent pre‐dispersal predation by insects such as bruchid beetles. By consuming pods early in the season and depositing seeds away from parent plants, megaherbivores may reduce beetle oviposition rates. Studies even suggest that seeds already predated upon may be sterilised in the gut while still viable (Coe and Coe 1987; Hoffman et al. 1989; Lamprey et al. 1974; Miller 1995; Miller and Coe 1993; Pellew and Southgate 1984).

During early seedling development (the first year of growth), megaherbivores may continue to shape recruitment outcomes through secondary effects. Dung deposition could offer a nutrient‐rich, moisture‐retaining microhabitat that can favour seedling emergence and early survival (Anderson et al. 2014; Miller and Coe 1993; Pellew and Southgate 1984). However, this is disputed by Miller (1995) who found lower seed germination rates in dung compared to bare soil. Additionally, depending on the plant species, seedling browsing by megaherbivores can have a negative effect on height growth and cause mortality, with consequences for population dynamics (Tsumele et al. 2007; Archibald and Bond 2003).

Despite growing recognition of megaherbivore–plant mutualisms (Awasthi et al. 2024; Campos‐Arceiz et al. 2008; Dinerstein and Wemmer 1988), research remains heavily skewed toward the African savanna elephant (*Loxodonta africana*; Bunney et al. 2017; Hyvarinen et al. 2021). The African black rhinoceros ( *Diceros bicornis* ) remains particularly understudied, with existing research largely focused on its seasonal dietary preferences and habitat use in cooler, more fertile regions (Hyvarinen et al. 2021; Landman et al. 2013; Lush et al. 2015; Schwabe et al. 2015; Shaw 2011; Sterk et al. 2023). Yet, due to their obligate browsing, seasonal frugivory and habitual defecation in fixed dung middens (Bunney et al. 2017) black rhinos may serve as important yet underappreciated facilitators of woody plant recruitment due to a possible megaherbivore–plant mutualism. Historically abundant across much of Africa (Owen‐Smith 1987, 1989), black rhinos may have exerted significant influence on vegetation dynamics in the past.

Here, we examined the effects of black rhinos on the germination and early recruitment of *Vachellia erioloba* , a widely distributed and ecologically important tree in arid regions (Odirile et al. 2019; Palgrave 1983; van Wyk and van Wyk 1997; Vincent et al. 2024). As a nitrogen‐fixer, *V. erioloba* improves soil fertility, provides shade and facilitates understorey plant growth, supporting hotspots of biodiversity (Scogings and Sankaran 2019; Seymour 2006; Vincent et al. 2024). *V. erioloba* invests in indehiscent pods (Miller 1995, 1996; Palgrave 1983; van Wyk and van Wyk 1997) and hard, semi‐water‐impermeable coats that delay germination (Baskin and Baskin 2004; Milton 1987; Odirile et al. 2019; Tran and Cavanagh 1984). Such protective structures pose a trade‐off; while enhancing seed longevity, they inhibit effective germination. For this reason, *V. erioloba* tends to have symbiotic relationships with large herbivores that consume pods and facilitate dormancy breakage (Seymour 2006). A range of large herbivores, including black rhino, kudu and giraffe, have been observed consuming *V. erioloba* pods. Yet, to date, only elephants have been empirically shown to enhance germination following gut passage (Coe and Coe 1987; Lamprey et al. 1974; Miller 1995; Pellew and Southgate 1984). We tested for a potential mutualism between black rhinos and *V. erioloba,* not only through germination enhancement but also by considering post‐germination seedling recruitment effects that are demographically critical in savanna environments. Few studies, if any, have explored such interactions at ontogenic stages other than germination.

Specifically, we hypothesised that:

1. Gut passage significantly enhances germination. We expected positive effects of scarification and weakening of the seed coat during gut passage would be reflected in the germinability of seeds collected from dung.
2. Seed viability is diminished by the duration between pod shedding and seed dormancy breakage. We expected that seeds from dung collected later in the season, which would have been retained in pods on the ground for a prolonged period, would be less viable than those ingested and dispersed before substantial exposure to environmental stressors.
3. Legacy effects of gut passage enhance the initial growth of seedlings. We expected acid scarification during digestion would improve seed coat permeability, enabling more efficient water uptake and faster resource allocation to initial root elongation and shoot emergence, resulting in enhanced seedling vigor.
4. Seedlings grown in dung recover better after simulated herbivory (clipping) than those grown in sand. We expected that properties of the dung, such as improved water retention or high nutrient content, would confer benefits to seedling development and thus enhance their resilience to disturbance events.

## 2 Materials and Methods

### 2.1 Study Site

This study took place at Tswalu Kalahari Private Game Reserve in the southern Kalahari Desert, Northern Cape, South Africa (27°13′30″ S and 22°28′40″ E). Summer temperatures exceed 40°C, and winter temperatures drop below freezing (Tokura et al. 2018). Rainfall is highly variable and arrives as short, patchy thunderstorms (van Rooyen and van Rooyen 2017, 2022). Elevation ranges from 1020 to 1586 m (Rymer et al. 2014; van Rooyen and van Rooyen 2017, 2022). Fire has been excluded since reserve establishment, aside from occasional accidental burns.

Vegetation is classified as Kalahari Dune Bushveld (Mucina and Rutherford 2006; van Rooyen and van Rooyen 2017, 2022), underlain by reddish‐brown aeolian sands (van Rooyen and van Rooyen 2017). Dominant woody species are patchily distributed (detailed in the Supporting Information, hereafter ‘SI’).

Tswalu hosts a range of large vertebrate herbivores native to the region, along with species that historically appeared seasonally but are now permanent residents within the fenced area (Webster et al. 2024). This includes a growing population of desert black rhino ( *Diceros bicornis bicornis* ), reintroduced from Etosha National Park in 1995. The southern Kalahari falls within their historic range, and they now serve as a flagship species for conservation at Tswalu.

### 2.2 Methods Overview

See Figure 1

**FIGURE 1:**
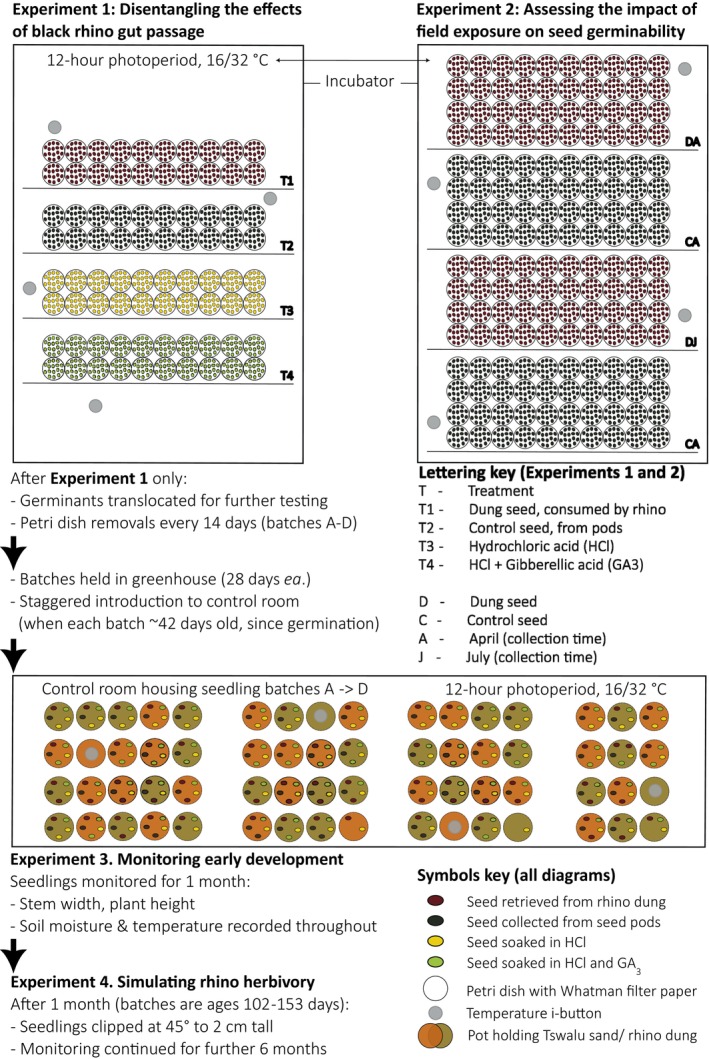
Overview of the experimental workflow, illustrating how surviving individuals were integrated into successive experiments testing germination, growth and recovery. Only Experiment 2 used a unique seed set.

### 2.3 Germination Assays

#### 2.3.1 Seed Collection

Seeds were initially collected from black rhino dung to identify the study species, followed by mature pod collection.

Dung samples were collected over 2 weeks each in April and July 2023, post‐wet season (November to March). By this stage, all indehiscent pod‐bearing species had produced mature pods, so their seeds would have been present in boluses if consumed by rhinos and able to withstand gut passage. The two collection periods were intended to contrast seeds with minimal (April) versus prolonged (July) environmental exposure. With assistance from field rangers, fresh black rhino dung (≤ 2 days) was located by backtracking from waterholes, and all intact seeds were removed from boluses. As rhinos have distinct territories, dung from a midden typically reflects the diet of only a few individuals. To maximise the number of individual rhinos sampled, thereby capturing variable gut passage effects on seeds, dung seeds were collected across Tswalu. This also optimised the genetic diversity among seeds to account for potentially varied germination responses.

Only *V. erioloba* seeds were found intact and in high densities (see SI). Accurate identification was confirmed by germinating and growing seedlings during the experiments until distinctive characteristics emerged.

In April, fallen pods were collected from beneath *V. erioloba* trees randomly distributed across Tswalu. Pods were diversely shaped and sized, particularly as the distance between trees increased (*pers. obs*.), which could be a morphological indication of genetic diversity, as found in *Vachellia caven* by Pometti et al. (2010).

Seeds were processed immediately at the C.L.I.ME Laboratory, Stellenbosch University (Climate and Invasions: Mechanisms in Ectotherms; Department of Botany and Zoology). Pods were opened and seeds submerged in distilled water to check for bruchid beetle infestation and viability. Only flawless, sinking seeds were selected as sample individuals. Dung seeds were rinsed with distilled water and processed similarly. By minimising variability in initial seed quality, processing standardised the viability potential between seed treatment groups, facilitating a robust comparison. The first germination trial commenced within a week but the second trial was delayed by 12 weeks due to incubator constraints, during which time seeds were stored at 22°C ± 2°C in paper bags.

#### 2.3.2 Microscope Assessment

Prior to the germination assays (Experiments 1 and 2), 10 seeds each from pods and dung were examined under a stereo microscope (LEICA M125, with MC190 HD camera) to check for visible differences in the external condition of the seed coats.

Refer to Figure 1 for a visual summary of experiments 1–4. Methodology is detailed below.

#### 2.3.3 Experiment 1: Disentangling the Effects of Gut Passage on Germination

To assess whether gut passage enhanced germinability, our first germination trial compared dung seeds to pod seeds. Only April‐collected seeds were used.

900 pod seeds and 300 dung seeds were disinfected in 3% chlorine bleach (1:5) for 2 min. Pod seeds were divided into three groups (*n* = 300 *ea*.): untreated Control, HCl‐treated and HCl + GA‐treated. To simulate acid scarification alone (disentangling it from other digestive processes like mechanical abrasion and exposure to heat or enzymes), the second group was soaked in 0.000033 M HCl for 20 h, followed by 0.00977 M HCl for another 20 h. Protocols were designed based on black rhino digestive information in Clemens and Maloiy (1982) and Dierenfield (1993). The third group was soaked in gibberellic acid (GA_3_; 50 mg/L, Kimix, ≥ 90% purity) for 8 h following similar HCl treatment. The control and GA groups served as minimum and maximum germination benchmarks, contextualising the dung and HCl treatments. See SI for a detailed explanation and the underlying rationale of the HCl and GA treatments.

The germination trial was run in a thermostatically controlled growth chamber (Panasonic MIR‐254). Each treatment group (Dung, Control, HCl and GA) comprised 20 replicate Petri dishes (9 cm), each lined with two layers of grade 1 Whatman filter paper, pre‐moistened with 5 mL of distilled water and containing 15 seeds. To prevent moisture loss, all Petri dishes were sealed with Parafilm M. The growth chamber was equipped with warm white fluorescent tubes (KD21 Striplight; Radiant, 16 W) and set to a fixed temperature regime of 16/32°C with a 12‐h thermoperiod and a matching light/dark photoperiod. Petri dishes were monitored daily for germinants (radicle ≥ 1 mm) over 86 days (~11 weeks), well beyond the typical germination period for *V. erioloba* seeds (6–8 weeks; Palgrave 1983). Moisture levels were maintained throughout. To avoid systematic effects related to positioning in the incubator, all dishes were re‐randomised every 2 days, as in Yang et al. (1999). Germinants were transplanted to seedling trays every 14 days.

#### 2.3.4 Experiment 2: Assessing Pod Exposure Effects on Seed Germinability

This trial compared the germinability of dung seeds collected in April and July. Both sets were from the same cohort, but July seeds had experienced several additional months of environmental exposure prior to ingestion by black rhinos. We hypothesised that July seeds would show reduced performance as a result, despite being visually intact.

We used 600 dung seeds from each month and 1200 pod seeds (April‐collected), divided into two control groups (*n* = 600 *ea*.). Each treatment (Dung April, Dung July, Control 1, Control 2) was sown in 40 replicate Petri dishes. Apart from the increased sample size, the experimental design replicated Experiment 1. The trial was terminated after 12 days due to time constraints linked to upcoming fieldwork.

### 2.4 Testing Early Seedling Growth and Resilience

#### 2.4.1 Experiment 3: Monitoring Early Seedling Development

Seedlings from Experiment 1 were used to assess whether gut passage conferred legacy effects on early seedling development. We expected Dung seedlings to grow faster and taller than Control seedlings.

Germinated seeds were removed from Petri dishes every 14 days and transplanted into seedling trays filled with builder's sand and perlite (1:1), a nutrient‐deficient medium used to avoid any confounding effects of substrate prior to the experiment. The trays were housed in a greenhouse with full‐spectrum daylight supplementation and relatively stable temperatures (16/32°C ± 5°C). Each seedling received 5 mL of distilled water weekly. After 28 days, seedlings were transplanted into 15 cm pots and moved to a climate‐controlled room for monitoring. This staggered schedule created four batches, each spaced ~14 days apart, that were all introduced at ~42 days of age.

Pots were filled with either Tswalu sand or fresh (≤ 2 days) black rhino dung, collected in the field and sealed in heavy‐duty bags for ≤ 2 weeks. Substrates were manually sifted to remove visible seeds. Each pot was layered with 5 cm of rockwool, 250 mL of perlite and topped with either sand or dung.

Seedlings from each germination treatment group (Control, Dung, HCl and GA) were assigned as evenly as possible to each substrate. At large, five seedlings were planted per pot. However, when the number of seedlings in a batch was not divisible by five, one pot contained fewer individuals to accommodate the remainder. This variation was unavoidable, as batch size was determined by the total number of seeds that germinated during the 14‐day window of the preceding germination trial. The variable *pot density* (i.e., the number of seedlings per pot) was recorded at planting and updated during biweekly monitoring to reflect any mortalities. Four additional pots (two per substrate) housed i‐Buttons at 1 cm depth to monitor soil temperature.

The room maintained a 16/32°C cycle and 12‐h photoperiod, lit by two full‐spectrum, 600 W LED plant ballasts (110 × 180 cm), delivering ~500 ppm. A malfunction spiked daytime temperatures to a maximum of 41°C for ~1 week, with minimal effect on nighttime temperatures. This short‐term fluctuation precisely replicated a Kalahari heat wave, which occurs frequently during the summer season we were simulating. Analysis revealed that seedling growth was unaffected.

Pots received 150 mL water twice weekly. Prior to watering, weekly soil moisture was recorded with an EC‐5 probe (ProCheck logger). Pots were re‐randomised biweekly. Plant growth measurements, namely plant length and basal stem diameter, were recorded every 2 weeks. Plant length was measured by tracing a string from the base of the cotyledons (where a scar remains after termination) to the apical bud and recording the string length. Stem diameter was measured using digital calipers below the first true leaves.

#### 2.4.2 Experiment 4: Clipping, to Simulate Black Rhino Herbivory

To assess whether rhino dung promotes development and confers resilience to disturbance, seedlings were clipped to 2 cm, after which their recovery in either substrate was monitored. We expected seedlings grown in dung to recover most effectively, as they could rely on substrate‐derived nutrients and moisture.

Plants were cleanly clipped with pruning shears at 45°, replicating the characteristic bite of a black rhino (Owen‐Smith 1988). Clipping prevents apical dominance and consequently induces branching, a common response to disturbance among *Acacias* in arid environments (Archibald and Bond 2003). To accommodate this, each branch length was traced with a string and summed to calculate total plant length. All other monitoring protocols matched those in Experiment 3.

All seedlings were clipped on the same day meaning that, since batches were staggered by ~14 days, there was a ~51‐day age gap between the oldest and youngest seedlings. This design allowed us to examine whether resilience to disturbance increased with seedling maturity. The full monitoring period, including early growth and post‐clipping recovery, lasted 93 days (±15 weeks).

### 2.5 Data Analysis

All statistical analyses in this study were conducted using the R statistical environment, version 4.4.2 (R Core Team 2024).

#### 2.5.1 Experiments 1 and 2: Disentangling the Effects of Gut Passage on Germination and Assessing the Impact of Field Exposure on Seed Germinability

We applied time‐to‐event models to analyse seed germination, as recommended by McNair et al. (2012) and Romano and Stevanato (2020), with methodological guidance from Onofri et al. (2010) and Scott et al. (1984). Unlike traditional germination methods (indices, ANOVAs, or linear regressions), time‐to‐event approaches model the distribution of individual germination events, rather than cumulative germination proportions, offering greater statistical power. We considered three main types of models: Kaplan–Meier (non‐parametric), Cox proportional‐hazards (semi‐parametric) and Accelerated Failure Time (parametric) models. We formatted germination data from either trial in binary format. After inspecting survival curves and log–log plots, we found the data exhibited a slightly leptokurtic distribution, so it violated parametric assumptions, particularly in the second trial. Consequently, the non‐parametric (Kaplan–Meier) and semi‐parametric (Cox proportional‐hazards) approaches were chosen for reporting in both analyses.

Models were run using the *survival* package (Therneau 2023; version 3.8‐1) and visualised with *survminer* (Kassambara and Kosinski 2021; version 0.4.9). Statistical significance was assessed using log‐rank (Kaplan–Meier) and Wald (Cox) tests, with *p*‐values reported and *α* = 0.05. The Kaplan–Meier log‐rank test models the survival function 𝑆(𝑡), or the probability of a seed not germinating at time 𝑡. This is the inverse of the cumulative incidence 𝐹(𝑡)/1−𝑆(𝑡), representing the instantaneous probability of germination. The Cox model estimates the hazard function 𝐻(𝑡), or the cumulative ‘risk’ of germination.

#### 2.5.2 Experiments 3 and 4: Monitoring Early Seedling Development and Clipping to Simulate Black Rhino Herbivory

Seedling growth in sand and dung substrates, pre‐ and post‐clipping, was analysed by means of Generalised Linear Mixed Models (GLMMs), using the *glmer* function (*Lme4* version 1.1‐35.4; Bates et al. 2015).

Basal stem diameter, converted to stem area (πr^2^), was selected as the main response variable because this measure demonstrated the best model fit. This aligns with findings that stem diameter scales with leaf biomass across a wide variety of tree species (Enquist and Niklas 2002; Sun et al. 2019). Plant growth data were separated into two datasets (pre‐ and post‐clipping) that were treated as separate experiments, so analysed independently but using the same statistical framework. Data from the first 18 days of monitoring were excluded from Experiment 3 because seedlings only had a radicle and cotyledons protruding from the seed, making stem‐related measurements unfeasible. Monitoring thus spanned 4 weeks for Experiment 3 (pre‐clipping) and 9 weeks for Experiment 4 (post‐clipping).

Prior to the main analysis, substrate differences in soil moisture and temperature were tested using Welch's *t*‐tests and collinearity among predictors was evaluated via Pearson's correlation matrices (*chart. Correlation* function; *PerformanceAnalytics* version 2.0.4; Peterson and Carl 2020). Variables with coefficients ≥ 0.75 were not jointly included in models. Model distributions were selected based on residual plots and fit diagnostics (*fitdistrplus* version 1.2‐1; Delignette‐Muller and Dutang 2015). Although the data were normally distributed, heteroscedasticity necessitated a semi‐parametric approach. The Gamma distribution provided the best fit for both datasets and was theoretically applicable since it accommodates positive, skewed and continuous data and is commonly used for repeated measures (Eric et al. 2021). Unlike the normal distribution (mean, SD), the Gamma distribution uses shape and scale parameters, which offer more flexibility in variance (Eric et al. 2021). We applied a log link, which too accommodates positive‐only data with positively skewed errors and can represent an underlying multiplicative process, which is common in ecology.

Candidate model selection was conducted to determine optimal model structure (Tables S1 and S2). While the experimental treatments were retained in all candidate models, the selection process considered the inclusion of additional biologically motivated covariates like soil moisture, temperature and pot density, as well as feasible interaction terms. Candidate models were ranked using the second‐order Akaike Information Criterion (AICc), computed via the *AICc* function (*AICcmodavg* version 2.3‐3; Mazerolle 2023). AICc was preferred over AIC due to small sample size, since it includes a correction for low numbers of observations relative to model parameters (Hurvich and Tsai 1989). Model rankings were based on ΔAICc values and cumulative Akaike weights (*aictab* function). Following Burnham and Anderson (2002), models with ΔAICc ≤ 2 were considered equally plausible; those with ΔAICc between 2 and 7 had less support; and those with ΔAICc > 7 were excluded from interpretation. If ΔAICc ≤ 2 between the top models, biological relevance was considered before selecting the final model. Multicollinearity among covariates was assessed using variance inflation factors (VIFs) or generalised VIFs (GVIFs) for models with interaction terms, via the *vif* function (*car* version 3.1‐2; Fox et al. 2019). We checked for overdispersion using the *dispersion_glmer* function (*blmeco* version 1.4; Korner‐Nievergelt et al. 2015). Marginal and conditional *R* ^2^ values were calculated via *r.squaredGLMM* (*MuMIn* version 1.48.4; Bartoń 2024).

## 3 Results

### 3.1 Germination Assays

#### 3.1.1 Microscope Assessment: Qualitative Results

**FIGURE 2:**
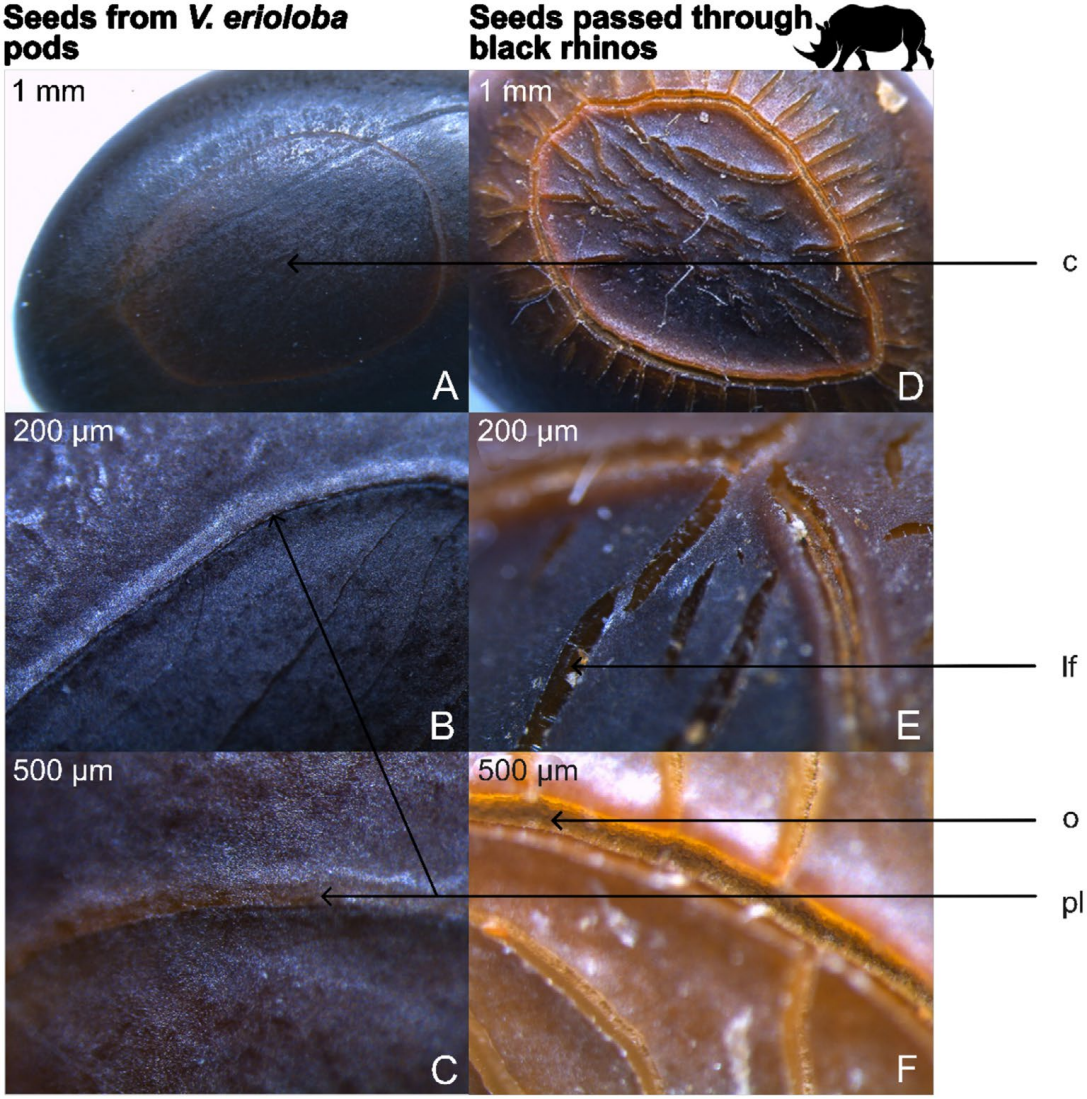
Stereo microscope images of *Vachellia erioloba* seeds from pods (A–C) and dung (D–F) at 1 mm, 200 μm and 500 μm. A–C: Seed coats are well sealed and physically dormant (pl—pleurogram; c—centre). D–F: Centre of seeds exhibit distinct linear fissures and the pleurogram has opened (lf—linear fissure; o—opening).

Stereo microscope inspection revealed that dung seeds consistently displayed distinct morphological differences from pod seeds. They were swollen, lighter in colour (light brown vs. dark black‐brown) and had a softened seed coat with linear fissures and an open pleurogram (Figure 2). In contrast, control seeds were smaller, darker and retained a hard, well‐sealed exterior.

#### 3.1.2 Experiment 1: Disentangling the Effects of Gut Passage on Germination

Germination differed significantly among treatments (Kaplan–Meier log‐rank test: *χ* ^2^ = 38.5, df = 3, *p* < 0.0001). Pairwise comparisons showed that Dung, HCl and GA treatments each differed from the Control but not from one another (Table S3).

**FIGURE 3:**
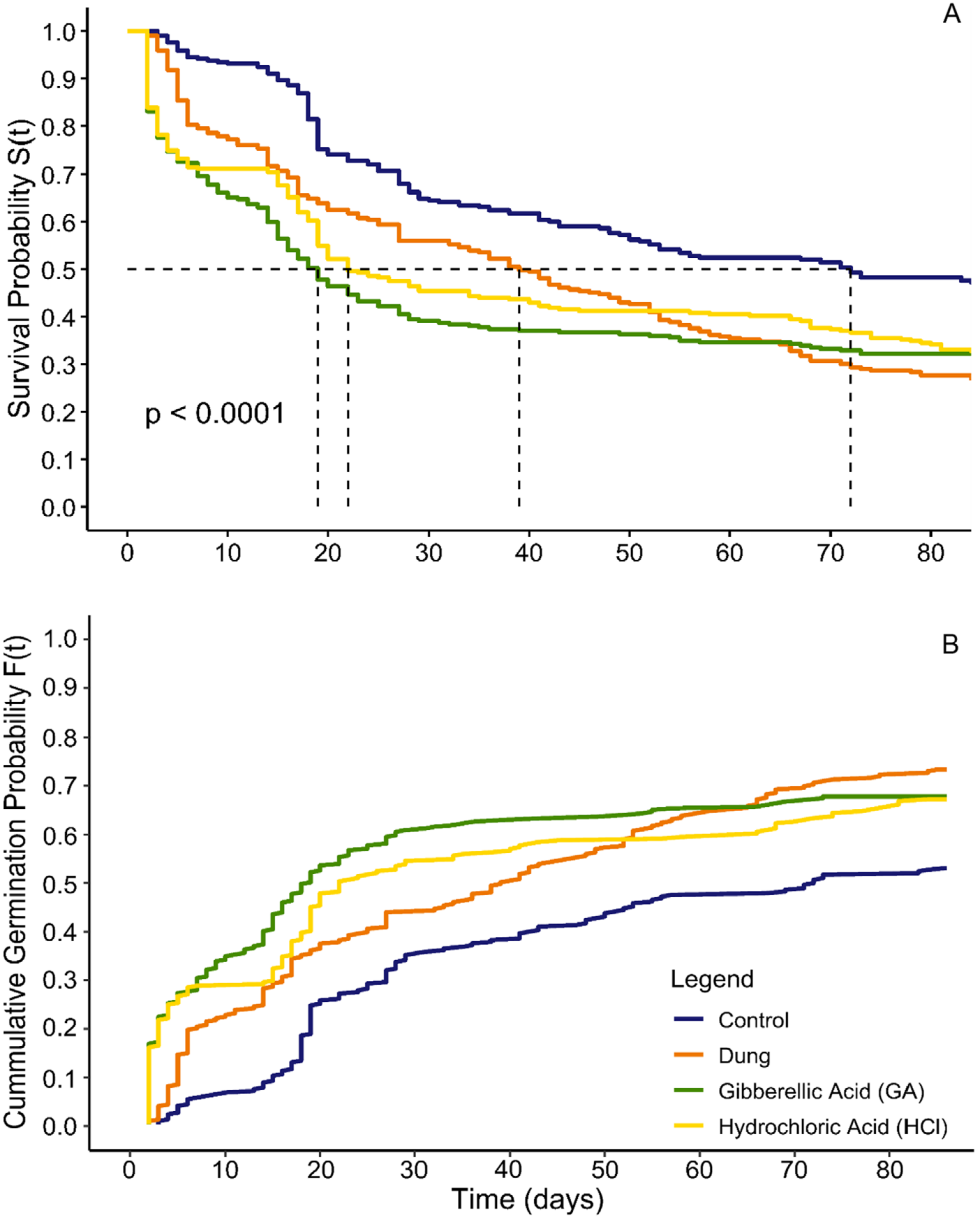
Kaplan–Meier (A) and cumulative incidence (B) curves for Control, Dung, Hydrochloric Acid and Gibberellic Acid seeds over ~86 days.

Although germination among the treatment groups was not statistically distinct, HCl and GA treatments showed early germination flushes that plateaued like the Control, whereas germination in the Dung group remained steady throughout, resulting in the highest total germinations (+40%) and longest duration (+13 days) relative to the Control (Figure 3).

Cox proportional‐hazards models confirmed these trends: all treatments had significantly higher germination risk than the Control, with GA highest (Cox PH, HCl: *z* = 4.74, Dung: *z* = 4.86, GA: *z* = 5.71, *p* < 0.0001; Table S4, Figure S1).

#### 3.1.3 Experiment 2: Assessing Pod Exposure Effects on Seed Germinability

Germination differed significantly among treatments (Kaplan–Meier log‐rank test: *χ* ^2^ = 169, df = 3, *p* < 0.0001). Pairwise comparisons showed that both Dung treatments differed significantly from both Control groups and from each other, while the Control treatments did not differ (Table S5).

**FIGURE 4:**
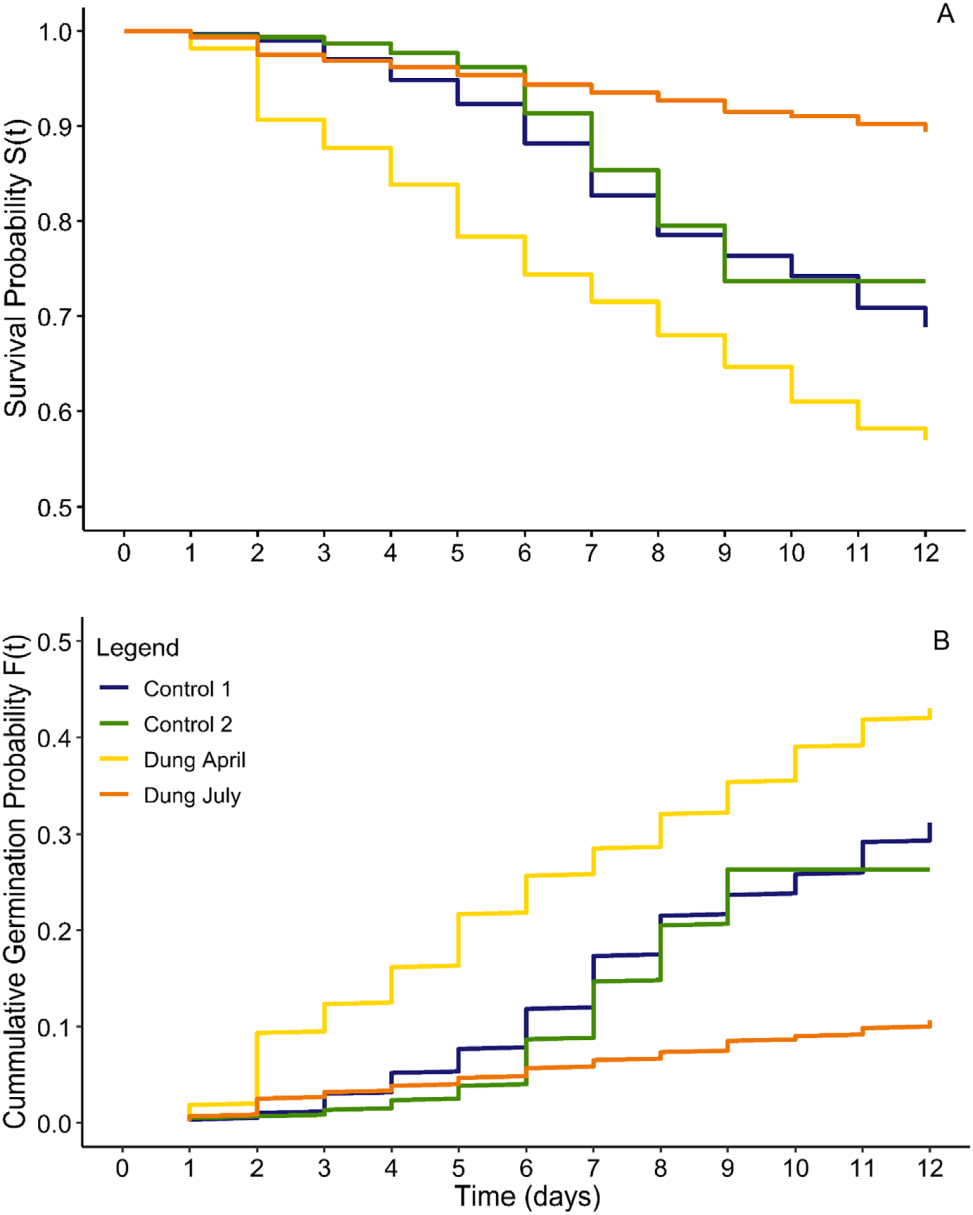
Kaplan–Meier (A) and cumulative incidence (B) curves for Controls and Dung seeds collected in April and July over 12 days.

Graphical outputs show April Dung seeds had the highest germinability, while July Dung seeds performed worst—even relative to controls (Figure 4). Due to the short trial duration, germination curves did not asymptote in all treatments.

Cox proportional‐hazards models corroborated these findings: April Dung seeds had a significantly higher hazard of germination (*z* = 4.80, *p* < 0.0001), and July Dung seeds had a significantly lower hazard (*z* = −8.07, *p* < 0.0001), relative to Control 1. Control 2 showed a slightly lower but non‐significant hazard than Control 1 (Table S6; Figure S2).

### 3.2 Testing Early Seedling Growth and Resilience

#### 3.2.1 Summary Statistics

**FIGURE 5:**
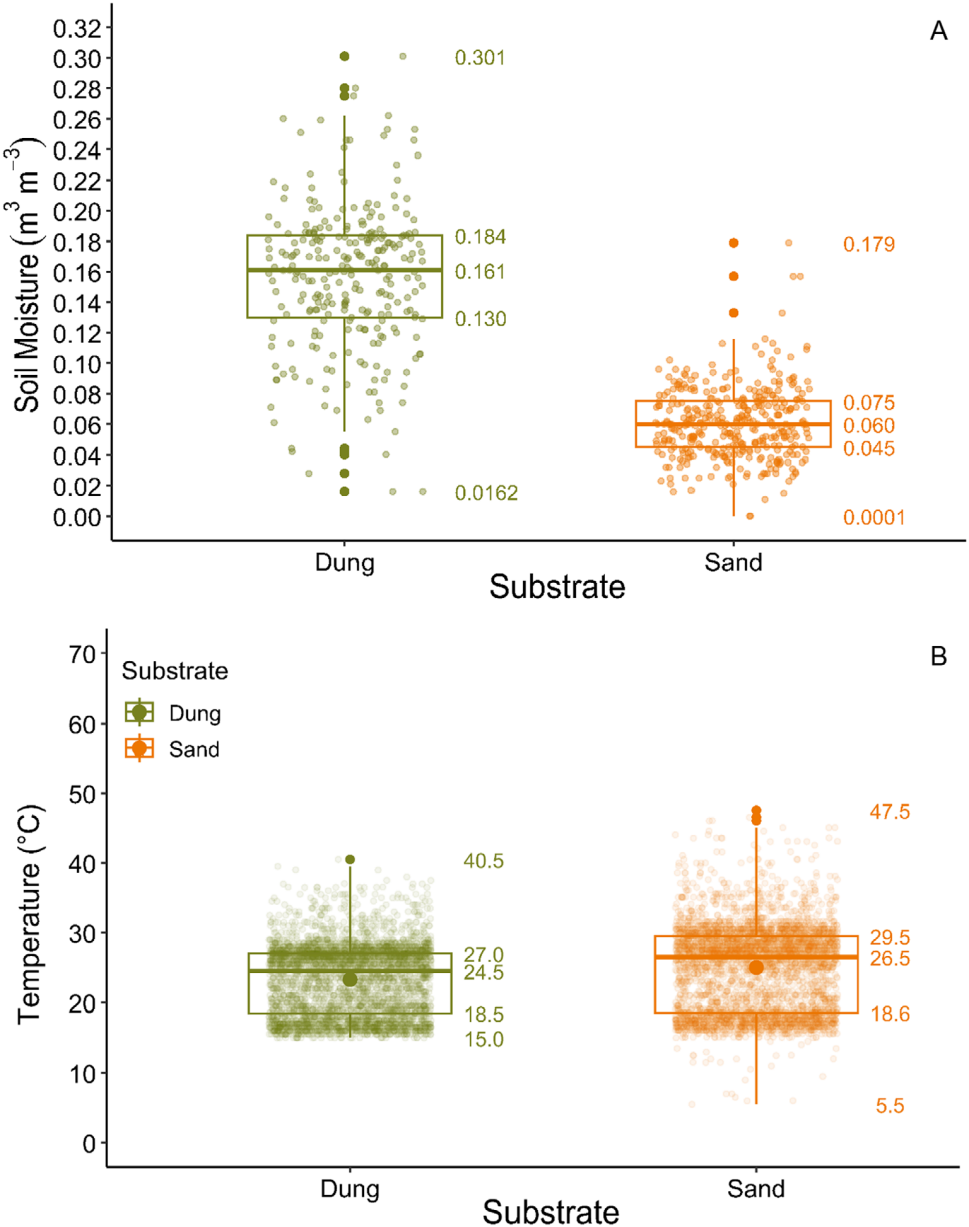
Boxplots of (A) soil moisture (m^3^m^−3^) and (B) soil temperature (°C) in Tswalu sand and black rhino dung.

Dung was significantly moister than sand (Welch's *t*‐test: *t* = 30.70, df = 360.26, *p* < 0.0001), with a mean difference of 0.0956 m^3^m^−3^ (Figure 5A). Sand was significantly warmer (Welch's *t*‐test: *t* = −7.03, df = 545.37, *p* < 0.0001), averaging 2°C higher than dung (Figure 5B).

#### 3.2.2 Experiment 3: Monitoring Early Seedling Development

A notable negative correlation was found between soil moisture and average maximum temperature (Pearson's: *r* = −0.71, *p* < 0.0001).

The final model showed no overdispersion (dispersion parameter = 0.486) and had acceptable explanatory power (*R*‐squared (trigamma): *R* ^2^_m_ = 0.453, *R* ^2^_c_ = 0.886), though individual variation in growth patterns remained high.

**FIGURE 6:**
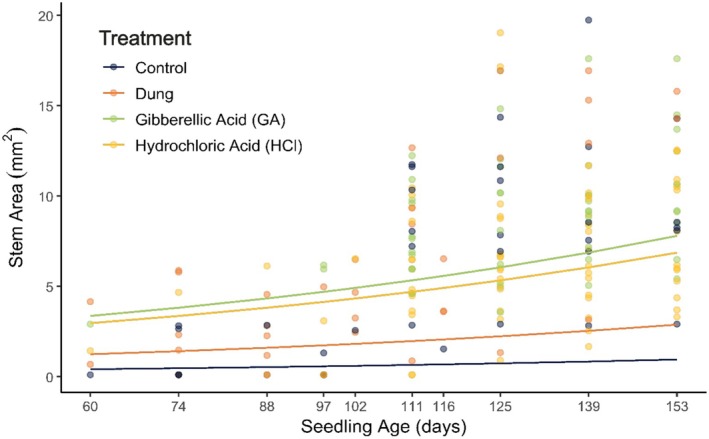
Predictive curves depicting early seedling growth (~60 to ~153 days) in climate‐controlled conditions.

Legacy effects from germination treatments were significant for Control (Wald: *z* = 2.57, *p* = 0.01), HCl (Wald: *z* = 3.36, *p* < 0.001) and GA (Wald: *z* = 3.53, *p* < 0.001). Although positive, effects from Dung (Wald: *z* = 1.68, *p* = 0.09) were insignificant (Figure 6, Table S7).

Seedling age positively affected growth (Wald: *z* = 3.81, *p* < 0.001). Contrastingly, soil moisture (Wald: *z* = −4.78, *p* < 0.00001) and pot density (Wald: *z* = −2.25, *p* = 0.02) negatively affected growth, while temperature was marginally insignificant (Wald: *z* = −1.91, *p* = 0.0562). A positive interaction was detected between soil moisture and pot density (Wald: *z* = 2.95, *p* < 0.01).

Mortality patterns varied across groups. From initial to final sample counts: Control (*n* = 31 → 7), Dung (24 → 8), HCl (19 → 17) and GA (18 → 12) over 5 weeks.

#### 3.2.3 Experiment 4: Clipping, to Simulate Black Rhino Herbivory

A moderate negative correlation was found between soil moisture and average maximum temperature (Pearson's: *r* = −0.54, *p* < 0.0001).

The final model showed no overdispersion (dispersion parameter =0.253) and had acceptable explanatory power (*R*‐squared (trigamma): *R* ^2^_m_ = 0.423, *R* ^2^_c_ = 0.700), though individual variation in growth patterns remained high.

**FIGURE 7:**
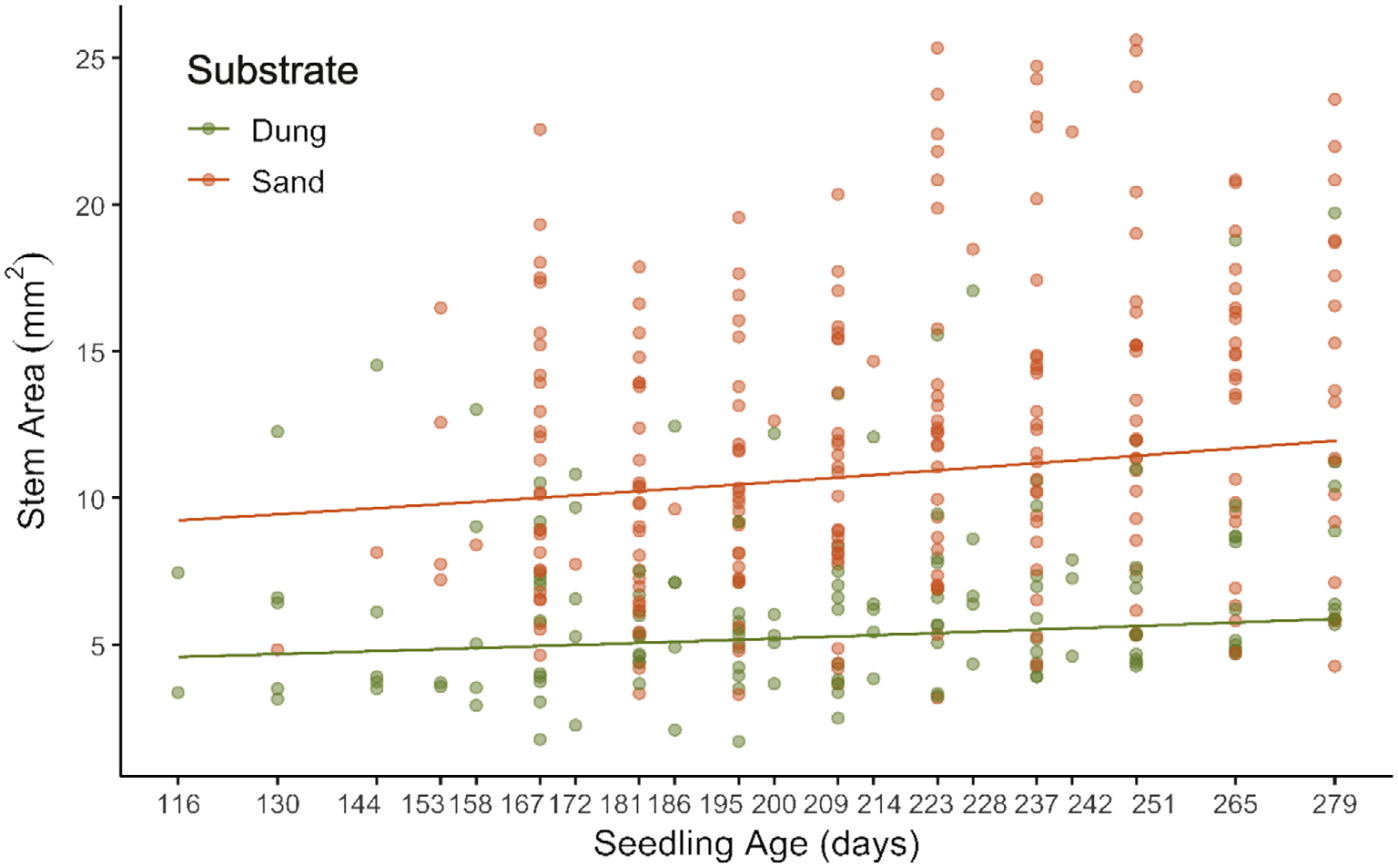
Predictive curves illustrating seedling recovery post‐clipping (~116 to ~279 days) in climate‐controlled conditions.

Dung had a strong positive effect on recovery (Wald: *z* = 8.30, *p* < 0.000001); however, sand showed the strongest positive effect (Wald: *z* = 4.01, *p* < 0.0001). Seedling age (days) significantly influenced recovery (Wald: *z* = 3.36, *p* < 0.001), with older individuals recovering best after clipping (Figure 7, Table S8).

Both temperature (Wald: *z* = 0.24, *p* = 0.81) and soil moisture (Wald: *z* = 1.35, *p* = 0.175) had insignificant effects on recovery. A negative interaction was detected between substrate and temperature; however, this was found to be marginally insignificant (Wald: *z* = −1.70, *p* = 0.0882).

Substrate had no effect on mortality, since sample sizes remained constant over 9 weeks (sand: *n* = 27; dung: *n* = 16); apart from one death in the sand group in the final week.

## 4 Discussion

This study demonstrates a significant mutualistic relationship between black rhinos and *V. erioloba* . The findings of the various experiments are not all consistent with the hypotheses initially proposed. The results suggest that this mutualism is not driven primarily by enhanced seedling development and recovery through legacy effects of gut passage (Hypothesis 3), nor potentially beneficial effects of growth in dung middens (Hypothesis 4) but instead stems from gut passage effects on germination (Hypothesis 1). In addition to increasing total germinations, gut passage both accelerates earlier germination and extends the total germination period, producing a seedling cohort with both older individuals and greater age variation. Such a population structure may enhance persistence beyond the germination bottleneck. This finding aligns with studies on other megaherbivores, which have demonstrated the role of elephants in facilitating seed germination (Bunney et al. 2017; Campos‐Arceiz and Blake 2011).

### 4.1 Black Rhino Increase Germination Rates and Germination Duration

Upon microscopic inspection (Figure 2), dung seeds displayed morphological changes indicative of seed coat weakening (see SI for detailed descriptions of pleurogram structure and pigmentation differences). This was tested in the first germination trial, in which all treatment groups outperformed the Control (Figure 3). The paralleled germination curves of the chemically scarified groups (HCl and GA) and the Control suggest that acid soaking primarily overcomes physiological dormancy, as described by Kumar et al. (2024) and Baskin and Baskin (2004). The initial flush of germinations and prolonged germination period in the Dung group indicate that a distinct mechanism alters the pattern of release. Rhino gut passage most likely degrades the seed coat (i.e., breaks physical dormancy) by means of supplementary stressors like mechanical abrasion, enzymes and heat (Clauss et al. 2005; Clauss and Hummel 2005). We observed that Dung seedlings more easily emerged from the testa, whereas many Control and acid scarified seedlings remained constricted, leading to chlorosis and mortality due to limited light access (*pers. obs*.). These findings highlight potential limitations of using chemical scarification as a proxy for herbivore digestion in germination studies.

The distinct germination pattern of the seedling cohort in the Dung group may confer a bet‐hedging advantage, particularly in a pulse‐driven, semi‐arid environment like the southern Kalahari (February et al. 2019): Increased total germinations create a larger seedling cohort, early emergence increases the number of seedlings that quickly reach an advanced developmental stage, and the longer germination period increases the diversity of individuals across plant stages. As a result, there is a greater probability that more seedlings are better placed to respond to disturbance events. For example, early emergence may help seeds escape bruchid beetle predation, a known constraint on *Vachellia* recruitment (Coe and Coe 1987; Hoffman et al. 1989; Lamprey et al. 1974; Miller 1995; Miller and Coe 1993; Pellew and Southgate 1984). Early emergence is also critical in savannas because dry seasons impose severe water stress and aboveground biomass is frequently removed by herbivory and fire (Scogings and Sankaran 2019).

### 4.2 Megaherbivores Mitigate Seed Viability Loss Under Environmental Stress

Although our conclusions are limited by the short duration of the second germination trial, the poor performance of Dung seeds collected months later, in July (Figure 4), suggests that viable individuals, including some of the genetically fittest, were lost over time due to environmental exposure of pods and greater impacts of seed predators such as bruchid beetles. This result lends support to the idea that herbivore‐mediated dispersal may allow more seeds to escape such stressors because pods are ingested and seeds are stimulated to germinate while being transported away from the parent plant, as described by Coe and Coe (1987), Lamprey et al. (1974) and Miller (1995).

### 4.3 Gut Passage Has Limited Effects on Early Seedling Development

The slight but non‐significant positive effect of gut passage on early seedling growth suggests limited legacy enhancement. However, scaled to a natural setting like in Tswalu, even a minor positive effect could improve the number of seedlings reaching maturity. To the best of our knowledge, the investigation into legacy effects of gut passage on early seedling development is novel, and further study is thus required before definitive inferences can be made.

### 4.4 Gut Passage Advances Seedling Age, Enhancing Herbivory Resilience

Herbivory is a key driver of woody plant dynamics in African savannas (Anderson et al. 2014). While some juveniles can compensate for biomass loss, others suffer dieback under additional stressors such as fire or drought (Tsumele et al. 2007). Understanding these responses is essential for evaluating juvenile plant resilience (Tsumele et al. 2007).

The finding that seedlings were adapted to both substrates but recovered slightly better in sand after simulating black rhino herbivory suggests that dung may not confer the expected benefits for seedling development. This result aligns with Miller (1995), who also reported a limited seedling response to faecal nutrients. As nitrogen‐fixing legumes (Vincent et al. 2024), *Vachellia* are likely well adapted to Tswalu's aeolian sands. However, since dung is typically deposited on sand and has moisture‐retaining properties, the sand beneath should remain wetter than exposed sand. In reality, seedlings would grow in a mix of both substrates—a condition that should be tested in future experiments. Future studies should also account for secondary dispersers like dung beetles, which may amplify or dilute dung effects on seedling growth by creating fine‐scale heterogeneity in nutrient microsites across the landscape (Veldhuis et al. 2018).

The key result was that older seedlings recovered most effectively from clipping. This resilience advantage likely stemmed from gut passage effects on germinability (Experiment 1), which increases the probability that more seedlings are better placed to respond to such disturbance events, as discussed in section 4.1. Thus, it is early maturation, not growth in dung, that promotes resilience and survival beyond the recruitment bottleneck.

### 4.5 Future Research

As with many gut‐passed germination studies, relying on intact seeds from faeces may introduce a positive bias in estimating germination benefits. Rogers et al. (2021) emphasise the need to quantify seeds ingested, survivors and those viable. Zoo‐based feeding trials, like those by Clauss et al. (2005) and Dierenfield (1993), can generate precise estimates. Extending germination assays to include seeds collected across full seasonal cycles and varied exposure durations would improve insights regarding viability loss over time. Growth experiments using a medium that isolates dung's moisture‐retaining properties from its nutrient, acid and enzymatic components could clarify its contribution to drought resilience. Notably, post‐experiment observations showed that seedlings grown in dung survived 8 weeks without watering, while those in sand perished (*pers. obs*.). Additionally, insights into seedling growth patterns in varied soil types (within rhinos broader distribution) could be valuable. To better quantify the facilitative role of black rhino, future studies should examine dispersal potential under field conditions, including browsing habits, seed consumption rates, bolus distribution and seasonal dispersal patterns across years of varying pod production. Long‐term field monitoring is also needed to determine whether initial germination gains translate into recruitment, since repeated defoliation and other stressors could hinder long‐term survival, as described in Scogings and Sankaran (2019). Applying these recommendations across woody species could inform a comprehensive model of rhino‐plant interactions to guide management strategies that integrate vegetative outcomes.

## 5 Conclusions

This study provides the first experimental evidence of a mutualistic interaction between black rhinoceroses and *V. erioloba* . By assessing effects across multiple early ontogenetic stages, we show that rhino gut passage shapes seedling cohort structure, increasing germinations and the number of older, more resilient individuals.

While our results establish a facilitative mechanism, the scale and broader ecological consequences remain uncertain. In the absence of elephants, black rhino are the largest browsing herbivores able to persist in fenced, arid reserves across southern Africa. As such, their contributions to seed dispersal and recruitment may meaningfully influence vegetation structure and community composition. Further research is needed to evaluate the ecological significance of this interaction under natural conditions.

This work addresses a longstanding bias toward African savanna elephants in megaherbivore research (Bunney et al. 2017; Hyvarinen et al. 2021). By identifying a new plant–herbivore mutualism, it also contributes to a more nuanced understanding of megaherbivores as both disturbance agents and plant facilitators in arid ecosystems.

## Author Contributions

**O. E. Jones:** conceptualisation (lead), data curation (lead), formal analysis (lead), funding acquisition (lead), investigation (lead), methodology (lead), project administration (lead), resources (supporting), software (lead), visualisation (lead), writing – original draft (lead), writing – review and editing (lead). **H. Beckett:** conceptualisation (supporting), data curation (supporting), formal analysis (supporting), investigation (supporting), methodology (supporting), project administration (supporting), supervision (lead), validation (lead), visualisation (supporting), writing – review and editing (supporting). **A. J. Abraham:** conceptualisation (supporting), data curation (supporting), formal analysis (supporting), investigation (supporting), methodology (supporting), project administration (supporting), supervision (lead), validation (lead), visualisation (supporting), writing – review and editing (lead). **N. P. Makunga:** conceptualisation (supporting), data curation (supporting), formal analysis (supporting), investigation (supporting), methodology (supporting), resources (supporting), supervision (supporting), validation (lead), visualisation (supporting), writing – review and editing (supporting). **G. F. Midgley:** conceptualisation (supporting), data curation (supporting), formal analysis (supporting), investigation (supporting), methodology (supporting), project administration (supporting), resources (lead), supervision (lead), validation (lead), visualisation (supporting), writing – review and editing (supporting).

## Conflicts of Interest

The authors declare no conflicts of interest.

## Supporting information


**Data S1:** ece371951‐sup‐0001‐supinfo.docx.

## Data Availability

All datasheets and coding scripts utilised for the experiments in this study are available on DRYAD, submitted with DOI https://doi.org/10.5061/dryad.9ghx3fftr.

## References

[ece371951-bib-0001] Abraham, A. J. , T. O. Prys‐Jones , A. De Cuyper , et al. 2020. “Improved Estimation of Gut Passage Time Considerably Affects Trait‐Based Dispersal Models.” Functional Ecology 35: 860–869. 10.1111/1365-2435.13726.

[ece371951-bib-0002] Anderson, T. M. , M. Schütz , and A. C. Risch . 2012. “Seed Germination Cues and the Importance of the Soil Seed Bank Across an Environmental Gradient in the Serengeti.” Oikos 121, no. 2: 306–312. 10.1111/j.1600-0706.2011.19803.x.

[ece371951-bib-0003] Anderson, T. M. , M. Schütz , and A. C. Risch . 2014. “Endozoochorous Seed Dispersal and Germination Strategies of Serengeti Plants.” Journal of Vegetation Science 25, no. 3: 636–647. 10.1111/jvs.12110.

[ece371951-bib-0004] Archibald, S. A. , and W. J. Bond . 2003. “Growing Tall vs Growing Wide: Tree Architecture and Allometry of *Acacia karroo* in Forest, Savanna, and Arid Environments.” Oikos 102, no. 1: 3–14. 10.1034/j.1600-0706.2003.12181.x.

[ece371951-bib-0005] Awasthi, B. , K. R. McConkey , N. Subedi , B. R. Lamichhane , S. T. Aluthwattha , and J. Chen . 2024. “Seed Dispersal Effectiveness by Greater One‐Horned Rhinos and Domestic Bovids of a Megafaunal Fruit.” Global Ecology and Conservation 54: 1–12. 10.1016/j.gecco.2024.e03120.

[ece371951-bib-0006] Bartoń, K. 2024. “MuMIn: Multi‐Model Inference.” Version 1.48.4. 10.32614/CRAN.package.MuMIn.

[ece371951-bib-0007] Baskin, C. C. , and J. M. Baskin . 2001. Seeds: Ecology, Biogeography, and Evolution of Dormancy and Germination (Repr.). Academic Press.

[ece371951-bib-0008] Baskin, J. M. , and C. C. Baskin . 2004. “A Classification System for Seed Dormancy.” Seed Science Research 14, no. 1: 1–16. 10.1079/SSR2003150.

[ece371951-bib-0009] Bates, D. , M. Mächler , B. Bolker , and S. Walker . 2015. “Fitting Linear Mixed‐Effects Models Using lme4.” Journal of Statistical Software 67, no. 1: 1–48. 10.18637/jss.v067.i01.

[ece371951-bib-0010] Bond, W. J. 2019. Open Ecosystems: Ecology and Evolution Beyond the Forest Edge. 1st ed. Oxford university press.

[ece371951-bib-0011] Bunney, K. , W. J. Bond , and M. Henley . 2017. “Seed Dispersal Kernel of the Largest Surviving Megaherbivore – The African Savanna Elephant.” Biotropica 49, no. 3: 395–401. 10.1111/btp.12423.

[ece371951-bib-0012] Burnham, K. P. , and D. R. Anderson . 2002. Model Selection and Multimodel Inference: A Practical Information‐Theoretic Approach. 2nd ed. Springer. 10.1007/b97636.

[ece371951-bib-0013] Campos‐Arceiz, A. , and S. Blake . 2011. “Megagardeners of the Forest – The Role of Elephants in Seed Dispersal.” Acta Oecologica 37, no. 6: 542–553. 10.1016/j.actao.2011.01.014.

[ece371951-bib-0014] Campos‐Arceiz, A. , A. R. Larrinaga , U. R. Weerasinghe , et al. 2008. “Behaviour Rather Than Diet Mediates Seasonal Differences in Seed Dispersal by Asian Elephants.” Ecology 89, no. 10: 2684–2691. 10.1890/07-1573.1. 18959306

[ece371951-bib-0015] Case, M. F. , C. Wigley‐Coetsee , N. Nzima , P. F. Scogings , and C. A. Staver . 2019. “Severe Drought Limits Trees in a Semi‐Arid Savanna.” Ecology 100, no. 11: e02842. 10.1002/ecy.2842. 31339179

[ece371951-bib-0016] Clark, J. S. 1998. “Why Trees Migrate So Fast: Confronting Theory With Dispersal Biology and the Paleorecord.” American Naturalist 152, no. 2: 204–224. 10.1086/286162. 18811386

[ece371951-bib-0017] Clauss, M. , T. Froeschle , J. Castell , et al. 2005. “Fluid and Particle Retention Times in the Black Rhinoceros, *Diceros bicornis*, a Large Hindgut‐Fermenting Browser.” Acta Theriologica 50, no. 3: 367–376. 10.1007/BF03192632.

[ece371951-bib-0018] Clauss, M. , and J. Hummel . 2005. “The Digestive Performance of Mammalian Herbivores: Why Big May Not Be That Much Better.” Mammal Review 35, no. 2: 174–187. 10.1111/j.1365-2907.2005.00062.x.

[ece371951-bib-0019] Clemens, E. T. , and E. M. O. Maloiy . 1982. “The Digestive Physiology of Three East African Herbivores: The Elephant, Rhinoceros and Hippopotamus.” Journal of Zoology 198: 141–156.

[ece371951-bib-0020] Coe, M. , and C. Coe . 1987. “Large Herbivores, *Acacia* Trees and Bruchid Beetles.” South African Journal of Science 83, no. 1: 624–635.

[ece371951-bib-0021] Delignette‐Muller, M. L. , and C. Dutang . 2015. “Fitdistrplus: An *R* Package for Fitting Distributions.” Journal of Statistical Software 64, no. 4: 1–34. 10.18637/jss.v064.i04.

[ece371951-bib-0022] Dierenfield, E. S. 1993. “Black Rhino Nutrition: An Overview.” Wildlife Conservation Society 1, no. 1: 10–12.

[ece371951-bib-0023] Dinerstein, E. , and C. M. Wemmer . 1988. “Fruits Rhinoceros Eat: Dispersal of *Trewia Nudiflora* (Euphorbiaceae) in Lowland Nepal.” Ecology 69, no. 6: 1768–1774. 10.2307/1941155.

[ece371951-bib-0024] Doughty, C. E. , A. Wolf , and Y. Malhi . 2013. “The Legacy of the Pleistocene Megafauna Extinctions on Nutrient Availability in Amazonia.” Nature Geoscience 6, no. 9: 761–764. 10.1038/ngeo1895.

[ece371951-bib-0025] Doughty, C. E. , A. Wolf , N. Morueta‐Holme , et al. 2016. “Megafauna Extinction, Tree Species Range Reduction, and Carbon Storage in Amazonian Forests.” Ecography 39, no. 2: 194–203. 10.1111/ecog.01587.

[ece371951-bib-0026] Enquist, B. J. , A. J. Abraham , M. B. J. Harfoot , Y. Malhi , and C. E. Doughty . 2020. “The Megabiota Are Disproportionately Important for Biosphere Functioning.” Nature Communications 11, no. 1: 699. 10.1038/s41467-020-14369-y. PMC7000713 32019918

[ece371951-bib-0027] Enquist, B. J. , and K. J. Niklas . 2002. “Global Allocation Rules for Patterns of Biomass Partitioning in Seed Plants.” Science 295, no. 5559: 1517–1520. 10.1126/science.1066360. 11859193

[ece371951-bib-0028] Eric, U. , M. O. O. Oti , and C. E. Francis . 2021. “A Study of Properties and Applications of Gamma Distribution.” African Journal of Mathematics and Statistics Studies 4, no. 2: 52–65. 10.52589/AJMSS-MR0DQ1DG.

[ece371951-bib-0029] February, E. C. , C. Coetsee , G. D. Cook , J. Ratnam , and B. Wigley . 2019. “Physiological Traits of Savanna Woody Species: Adaptations to Resource Availability.” In Savanna Woody Plants and Large Herbivores, 1st ed., 309–329. John Wiley & Sons Ltd. 10.1002/9781119081111.ch11.

[ece371951-bib-0030] Fox, J. , S. Weisberg , and B. Price . 2019. “Car: Companion to Applied Regression. Version 3.1–3.” https://CRAN.R‐project.org/package=car.

[ece371951-bib-0031] Frei, E. R. , E. Bianchi , G. Bernareggi , et al. 2018. “Biotic and Abiotic Drivers of Tree Seedling Recruitment Across an Alpine Treeline Ecotone.” Scientific Reports 8, no. 10894: 1–12. 10.1038/s41598-018-28808-w. 30022032 PMC6052039

[ece371951-bib-0032] Fricke, E. C. , A. Ordonez , H. S. Rogers , and J.‐C. Svenning . 2022. “The Effects of Defaunation on Plants' Capacity to Track Climate Change.” Science 375, no. 6577: 210–214. 10.1126/science.abk3510. 35025640

[ece371951-bib-0033] García Criado, M. , I. H. Myers‐Smith , A. D. Bjorkman , C. E. R. Lehmann , and N. Stevens . 2020. “Woody Plant Encroachment Intensifies Under Climate Change Across Tundra and Savanna Biomes.” Global Ecology and Biogeography 29, no. 5: 925–943. 10.1111/geb.13072.

[ece371951-bib-0034] Gill, J. L. , J. W. Williams , S. T. Jackson , K. B. Lininger , and G. S. Robinson . 2009. “Pleistocene Megafaunal Collapse, Novel Plant Communities, and Enhanced Fire Regimes in North America.” Science 326, no. 5956: 1100–1103. 10.1126/science.1179504. 19965426

[ece371951-bib-0035] Haynes, G. 2012. “Elephants (And Extinct Relatives) as Earth‐Movers and Ecosystem Engineers.” Geomorphology 157: 99–107. 10.1016/j.geomorph.2011.04.045.

[ece371951-bib-0036] Hempson, G. P. , S. A. Archibald , and W. J. Bond . 2015. “A Continent‐Wide Assessment of the Form and Intensity of Large Mammal Herbivory in Africa.” Science 350, no. 6264: 1056–1061. 10.1126/science.aac7978. 26612946

[ece371951-bib-0037] Hobbs, N. T. 1996. “Modification of Ecosystems by Ungulates.” Journal of Wildlife Management 60, no. 4: 695. 10.2307/3802368.

[ece371951-bib-0038] Hoffman, M. T. , R. M. Cowling , C. Douie , and S. M. Pierce . 1989. “Seed Predation and Germination of *Acacia erioloba* in the Kuiseb River Valley, Namib Desert.” South African Journal of Botany 55, no. 1: 103–106. 10.1016/S0254-6299(16)31237-6.

[ece371951-bib-0039] Holdo, R. M. , R. D. Holt , and J. M. Fryxell . 2013. “Herbivore–Vegetation Feedbacks Can Expand the Range of Savanna Persistence: Insights From a Simple Theoretical Model.” Oikos 122, no. 3: 441–453. 10.1111/j.1600-0706.2012.20735.x.

[ece371951-bib-0040] Huang, Y. H. , N. Owen‐Smith , M. D. Henley , et al. 2023. “Variation in Herbivore Space Use: Comparing Two Savanna Ecosystems With Different anthrax Outbreak Patterns in Southern Africa.” Movement Ecology 11, no. 1: 46. 10.1186/s40462-023-00385-2. 37525286 PMC10392021

[ece371951-bib-0041] Hurvich, C. M. , and C.‐L. Tsai . 1989. “Regression and Time Series Model Selection in Small Samples.” Biometrika 76, no. 2: 297–307. 10.1093/biomet/76.2.297.

[ece371951-bib-0042] Hyvarinen, O. , M. Te Beest , E. Le Roux , et al. 2021. “Megaherbivore Impacts on Ecosystem and Earth System Functioning: The Current State of the Science.” Ecography 44, no. 11: 1579–1594. 10.1111/ecog.05703.

[ece371951-bib-0043] Kassambara, A. , and M. Kosinski . 2021. “Survminer: Drawing Survival Curves Using ‘ggplot2’. Version 0.4.9.” https://CRAN.R‐project.org/package=survminer.

[ece371951-bib-0044] Korner‐Nievergelt, F. , T. Roth , S. von Felten , J. Guelat , B. Almasi , and P. Korner‐Nievergelt . 2015. Bayesian Data Analysis in Ecology Using Linear Models With R, BUGS and Stan. Elsevier.

[ece371951-bib-0045] Kristensen, J. Å. , L. Barbero‐Palacios , I. C. Barrio , et al. 2024. “Tree Planting Is no Climate Solution at Northern High Latitudes.” Nature Geoscience 17, no. 11: 1087–1092. 10.1038/s41561-024-01573-4.

[ece371951-bib-0046] Kumar, M. , S. Sarvade , R. Kumar , and A. Kumar . 2024. “Pre‐Sowing Treatments on Seeds of Forest Tree Species to Overcome the Germination Problems.” Asian Journal of Environment & Ecology 23, no. 5: 1–18. 10.9734/ajee/2024/v23i5543.

[ece371951-bib-0047] Lamprey, H. F. , G. Halevy , and S. Makacha . 1974. “Interactions Between *Acacia*, Bruchid Seed Beetles and Large Herbivores.” African Journal of Ecology 12, no. 1: 81–85. 10.1111/j.1365-2028.1974.tb00109.x.

[ece371951-bib-0048] Landman, M. , D. S. Schoeman , and G. I. H. Kerley . 2013. “Shift in Black Rhinoceros Diet in the Presence of Elephant: Evidence for Competition?” PLoS One 8, no. 7: e69771. 10.1371/journal.pone.0069771. 23874997 PMC3714249

[ece371951-bib-0049] Lush, L. , M. Mulama , and M. Jones . 2015. “Predicting the Habitat Usage of African Black Rhinoceros ( *Diceros bicornis* ) Using Random Forest Models.” African Journal of Ecology 53, no. 3: 346–354. 10.1111/aje.12192.

[ece371951-bib-0050] Malhi, Y. , C. E. Doughty , M. Galetti , F. A. Smith , J.‐C. Svenning , and J. W. Terborgh . 2016. “Megafauna and Ecosystem Function From the Pleistocene to the Anthropocene.” Proceedings of the National Academy of Sciences 113, no. 4: 838–846. 10.1073/pnas.1502540113. PMC4743772 26811442

[ece371951-bib-0051] Mazerolle, M. J. 2023. “AICcmodavg: Model Selection and Multimodel Inference Based on AIC(c).” Version 2.3‐3. 10.32614/CRAN.package.AICcmodavg.

[ece371951-bib-0052] McNair, J. N. , A. Sunkara , and D. Frobish . 2012. “How to Analyse Seed Germination Data Using Statistical Time‐To‐Event Analysis: Non‐Parametric and Semi‐Parametric Methods.” Seed Science Research 22, no. 2: 77–95. 10.1017/S0960258511000547.

[ece371951-bib-0053] Midgley, G. F. , and W. J. Bond . 2015. “Future of African Terrestrial Biodiversity and Ecosystems Under Anthropogenic Climate Change.” Nature Climate Change 5, no. 9: 823–829. 10.1038/nclimate2753.

[ece371951-bib-0054] Midgley, J. J. , B. W. T. Coetzee , D. Tye , and L. M. Kruger . 2020. “Mass Sterilisation of a Common Palm Species by Elephants in Kruger National Park, South Africa.” Scientific Reports 10, no. 1: 11719. 10.1038/s41598-020-68679-8. 32678201 PMC7366642

[ece371951-bib-0055] Miller, M. F. 1995. “ *Acacia* Seed Survival, Seed Germination and Seedling Growth Following Pod Consumption by Large Herbivores and Seed Chewing by Rodents.” African Journal of Ecology 33, no. 3: 194–210. 10.1111/j.1365-2028.1995.tb00797.x.

[ece371951-bib-0056] Miller, M. F. 1996. “Dispersal of *Acacia* Seeds by Ungulates and Ostriches in an African Savanna.” Journal of Tropical Ecology 12, no. 3: 345–356. 10.1017/S0266467400009548.

[ece371951-bib-0057] Miller, M. F. , and M. Coe . 1993. “Is It Advantageous for *Acacia* Seeds to Be Eaten by Ungulates?” Oikos 66, no. 2: 364. 10.2307/3544827.

[ece371951-bib-0058] Milton, S. J. 1987. “Phenology of Seven *Acacia* Species in South Africa.” South African Journal of Wildlife Research 17, no. 1: 1–6.

[ece371951-bib-0059] Mucina, L. , and M. C. Rutherford , eds. 2006. The Vegetation of South Africa, Lesotho and Swaziland. South African National Biodiversity Institute.

[ece371951-bib-0060] Münch, Z. , L. Gibson , and A. Palmer . 2019. “Monitoring Effects of Land Cover Change on Biophysical Drivers in Rangelands Using Albedo.” Land 8, no. 2: 33. 10.3390/land8020033.

[ece371951-bib-0061] O'Connor, T. G. , J. R. Puttick , and M. T. Hoffman . 2014. “Bush Encroachment in Southern Africa: Changes and Causes.” African Journal of Range and Forage Science 31, no. 2: 67–88. 10.2989/10220119.2014.939996.

[ece371951-bib-0062] Odirile, O. , W. Mojeremane , D. Teketay , and K. Keotshepile . 2019. “Responses of Seeds of *Vachellia Erioloba* (E. Mey) P.J.H. Hurter in Botswana to Different Pre‐Sowing Treatment Methods.” International Journal of Biology and Biotechnology 16, no. 1: 181–188.

[ece371951-bib-0063] Onofri, A. , F. Gresta , and F. Tei . 2010. “A New Method for the Analysis of Germination and Emergence Data of Weed Species.” Weed Research 50, no. 3: 187–198. 10.1111/j.1365-3180.2010.00776.x.

[ece371951-bib-0064] Owen‐Smith, N. 1987. “Pleistocene Extinctions: The Pivotal Role of Megaherbivores.” Paleobiology 13, no. 3: 351–362. 10.1017/S0094837300008927.

[ece371951-bib-0065] Owen‐Smith, N. 1988. Megaherbivores: The Influence of Very Large Body Size on Ecology. Cambridge University Press.

[ece371951-bib-0066] Owen‐Smith, N. 1989. “Megafaunal Extinctions: The Conservation Message From 11,000 Years B.P.” Conservation Biology 3, no. 4: 405–412. 21129027 10.1111/j.1523-1739.1989.tb00246.x

[ece371951-bib-0067] Palgrave, K. C. 1983. Trees of Southern Africa. 2nd ed. Struik Publishers.

[ece371951-bib-0068] Parr, C. L. , C. E. R. Lehmann , W. J. Bond , W. A. Hoffmann , and A. N. Andersen . 2014. “Tropical Grassy Biomes: Misunderstood, Neglected, and Under Threat.” Trends in Ecology & Evolution 29, no. 4: 4. 10.1016/j.tree.2014.02.004. 24629721

[ece371951-bib-0069] Pellew, R. A. , and B. J. Southgate . 1984. “The Parasitism of *Acacia tortilis* Seeds in the Serengeti.” African Journal of Ecology 22, no. 1: 73–75. 10.1111/j.1365-2028.1984.tb00679.x.

[ece371951-bib-0070] Peterson, B. G. , and P. Carl . 2020. “PerformanceAnalytics: Econometric Tools for Performance and Risk Analysis. Version 2.0.4.” 10.32614/CRAN.package.PerformanceAnalytics.

[ece371951-bib-0071] Pires, M. M. , P. R. Guimarães , M. Galetti , and P. Jordano . 2018. “Pleistocene Megafaunal Extinctions and the Functional Loss of Long‐Distance Seed‐Dispersal Services.” Ecography 41, no. 1: 153–163. 10.1111/ecog.03163.

[ece371951-bib-0072] Pometti, C. L. , J. C. Vilardi , A. M. Cialdella , and B. O. Saidman . 2010. “Genetic Diversity Among the Six Varieties of *Acacia caven* (Leguminosae, Mimosoideae) Evaluated at the Molecular and Phenotypic Levels.” Plant Systematics and Evolution 284, no. 3–4: 187–199. 10.1007/s00606-009-0244-y.

[ece371951-bib-0073] R Core Team . 2024. R: A Language and Environment for Statistical Computing. Version 4.4.2. R Foundation for Statistical Computing. https://www.R‐project.org/.

[ece371951-bib-0074] Ripple, W. J. , T. M. Newsome , C. Wolf , et al. 2015. “Collapse of the World's Largest Herbivores.” Science Advances 1, no. 4: 1–12. 10.1126/sciadv.1400103. PMC4640652 26601172

[ece371951-bib-0075] Rogers, H. S. , B. R. Cavazos , A. M. Gawel , et al. 2021. “Frugivore Gut Passage Increases Seed Germination: An Updated Meta‐Analysis.” *bioRxiv*. 10.1101/2021.10.12.462022.

[ece371951-bib-0076] Romano, A. , and P. Stevanato . 2020. “Germination Data Analysis by Time‐To‐Event Approaches.” Plants 9, no. 5: 617. 10.3390/plants9050617. 32408713 PMC7285257

[ece371951-bib-0077] Rymer, T. L. , R. L. Thomson , and M. J. Whiting . 2014. “At Home With the Birds: Kalahari Tree Skinks Associate With Sociable Weaver Nests Despite African Pygmy Falcon Presence.” Austral Ecology 39, no. 7: 839–847. 10.1111/aec.12152.

[ece371951-bib-0078] Scholes, R. J. 2020. “The Future of Semi‐Arid Regions: A Weak Fabric Unravels.” Climate 8, no. 3: 43. 10.3390/cli8030043.

[ece371951-bib-0079] Schwabe, F. , T. Göttert , N. Starik , S. R. Levick , and U. Zeller . 2015. “A Study on the Postrelease Behaviour and Habitat Preferences of Black Rhinos ( *Diceros bicornis* ) Reintroduced Into a Fenced Reserve in Namibia.” African Journal of Ecology 53, no. 4: 531–539. 10.1111/aje.12245.

[ece371951-bib-0080] Scogings, P. F. , and M. Sankaran . 2019. “Woody Plants and Large Herbivores in Savannas: Ancient Past – Uncertain Future.” In Savanna Woody Plants and Large Herbivores, edited by P. F. Scogings and M. Sankaran , 683–712. Wiley. 10.1002/9781119081111.ch21.

[ece371951-bib-0081] Scott, S. J. , R. A. Jones , and W. A. Williams . 1984. “Review of Data Analysis Methods for Seed Germination.” Crop Science 24, no. 6: 1192–1199. 10.2135/cropsci1984.0011183X002400060043x.

[ece371951-bib-0082] Seymour, C. 2006. “The Influence of Size and Density of the Camelthorn ( *Acacia erioloba* Meyer) on Its Keystone Role in the Xeric Kalahari.” PhD thesis. University of Cape Town.

[ece371951-bib-0083] Shaw, J. A. 2011. “Adaptive Resource Use in a Re‐Introduced Black Rhinocerous Population.” PhD thesis. University of the Witwatersrand.

[ece371951-bib-0084] Smit, I. P. J. , and H. H. T. Prins . 2015. “Predicting the Effects of Woody Encroachment on Mammal Communities, Grazing Biomass and Fire Frequency in African Savannas.” PLoS One 10, no. 9: e0137857. 10.1371/journal.pone.0137857. 26379249 PMC4574768

[ece371951-bib-0085] Stavi, I. , T. A. Zinnes , A. Joseph , E. Solowey , and E. Groner . 2015. “The Role of Large Herbivores in Recruitment of *Acacia* Trees via Endozoochory in the Arava Valley, Israel.” European Journal of Wildlife Research 61, no. 5: 775–781. 10.1007/s10344-015-0954-0.

[ece371951-bib-0086] Sterk, M. , F. S. Cubas , B. Reinhard , F. Reinhard , K. Kleopas , and Z. Jewell . 2023. “The Importance of Large Pans and Surrounding Bushveld for Black Rhino (*Diceros bicornis* ssp. *Bicornis*) Habitat Use in the Kalahari: Implications for Reintroduction and Range Expansion.” Namibian Journal of Environment 7: 1–13.

[ece371951-bib-0087] Stevens, N. , C. E. R. Lehmann , B. P. Murphy , and G. Durigan . 2017. “Savanna Woody Encroachment Is Widespread Across Three Continents.” Global Change Biology 23, no. 1: 235–244. 10.1111/gcb.13409. 27371937

[ece371951-bib-0088] Sun, J. , M. Wang , M. Lyu , et al. 2019. “Stem Diameter (And Not Length) Limits Twig Leaf Biomass.” Frontiers in Plant Science 10: 185. 10.3389/fpls.2019.00185. 30846996 PMC6393343

[ece371951-bib-0089] Therneau, T. M. 2023. “A Package for Survival Analysis in R. Version 3.8‐1.” https://CRAN.R‐project.org/package=survival.

[ece371951-bib-0090] Tokura, W. , S. L. Jack , T. Anderson , and M. T. Hoffman . 2018. “Long‐Term Variability in Vegetation Productivity in Relation to Rainfall, Herbivory and Fire in Tswalu Kalahari Reserve.” Koedoe 60, no. 1: 1. 10.4102/koedoe.v60i1.1473.

[ece371951-bib-0091] Tran, V. N. , and A. K. Cavanagh . 1984. “Structural Aspects of Dormancy.” In Germination and Reserve Mobilisation, 1–44. Elsevier. 10.1016/B978-0-12-511902-3.50006-3.

[ece371951-bib-0092] Tsumele, J. , D. Mlambo , and A. Sebata . 2007. “Responses of Three *Acacia* Species to Simulated Herbivory in a Semi‐Arid Southern African Savanna.” African Journal of Ecology 45, no. 3: 324–326. 10.1111/j.1365-2028.2006.00713.x.

[ece371951-bib-0093] van Rooyen, G. , and N. van Rooyen . 2017. Ecological Evaluation of Tswalu Kalahari Reserve. Ekotrust.

[ece371951-bib-0094] van Rooyen, G. , and N. van Rooyen . 2022. Veld Condition Assessment of Tswalu Kalahari Reserve: Herbaceous Layer. Ekotrust.

[ece371951-bib-0095] van Wyk, B. , and P. van Wyk . 1997. Field Guide to Trees of Southern Africa. Struik Publishers.

[ece371951-bib-0096] Veldhuis, M. P. , M. I. Gommers , H. Olff , and M. P. Berg . 2018. “Spatial Redistribution of Nutrients by Large Herbivores and Dung Beetles in a Savanna Ecosystem.” Journal of Ecology 106, no. 1: 422–433. 10.1111/1365-2745.12874.

[ece371951-bib-0097] Vincent, B. , J. Bourillon , K. Gotty , et al. 2024. “Ecological Aspects and Relationships of the Emblematic *Vachellia* spp. Exposed to Anthropic Pressures and Parasitism in Natural Hyper‐Arid Ecosystems: Ethnobotanical Elements, Morphology, and Biological Nitrogen Fixation.” Planta 259, no. 6: 132. 10.1007/s00425-024-04407-0. 38662123 PMC11045644

[ece371951-bib-0098] Webster, A. B. , A. J. Abraham , and O. E. Jones . 2024. An Assessment of Large Herbivore Population Abundance and Demographics at Tswalu Kalahari Reserve: 2024 Update. University of Pretoria, Mammal Research Institute.

[ece371951-bib-0100] Yang, J. , J. Lovett‐Doust , and L. Lovett‐Doust . 1999. “Seed Germination Patterns in Green Dragon (Arisaema dracontium, Araceae).” American Journal of Botany 86, no. 8: 1160–1167. 10.2307/2656980. 10449396

[ece371951-bib-0099] Zhu, J. , N. Lukić , J. Pagel , and F. M. Schurr . 2023. “Density Dependence of Seed Dispersal and Fecundity Profoundly Alters the Spread Dynamics of Plant Populations.” Journal of Ecology 111, no. 8: 1735–1748. 10.1111/1365-2745.14142.

