# Supplementary material for: Endozoochory by Black Rhinoceroses Enhances Germination of a Key Arid Savanna Tree Species
Source: Ecol Evol. 2025 Sep 1;15(9):e71951. doi: 10.1002/ece3.71951 (PMC12401563; doi:10.1002/ece3.71951)
Supplement: Supplementary file 1 — Data S1: ece371951‐sup‐0001‐supinfo.docx. [file ECE3-15-e71951-s001.docx]

**Supporting Information**

**2. Methods**

*2.1 Study site*

Woody species are patchily distributed throughout Tswalu and dominated by blackthorns (*Senegalia mellifera* *subsp. Detinens*; Burch. Kyal. & Boatwr.; Fabaceae), camelthorns (*Vachellia erioloba*; E.Mey. P.J.H.Hurter; Fabaceae), shepherd's trees (*Boscia albitrunca*; Burch. Gilg & Gilg-Ben.; Capparaceae), grey camelthorns (Vachellia haematoxylon; Willd. Seigler & Ebinger; Fabaceae), the velvet raisin (*Grewia flava;* DC; Malvaceae) and the driedoring (*Rhigozum trichotomum*; Burch.; Bignoniaceae) (van Rooyen & van Rooyen, 2022). Woody species that occur in much lower densities include the candlepod acacia (*Vachellia hebeclada subsp. Hebeclada*; DC. Kyal. & Boatwr.; Fabaceae) and two confirmed *Lycium* species*,* namely *L.* *villosum (*Schinz; Solanaceae*)* and *L.* *cinereum* (Thunb.; Solanaceae) (van Rooyen & van Rooyen, 2022).

*2.3 Germination Assays*

*Experiment 1: Disentangling the effects of gut passage on germination*

Details of seed retrieval from black rhino dung:

It is important to contextualize our findings within the framework of seed types and primary dispersal mechanisms of the dominant woody species at Tswalu: As fruiting woody plants, *Lycium* species and *B. albitrunca* produce berries, while *G. flava* produces drupes (Palgrave, 1983; van Wyk & van Wyk, 1997). All are believed to be bird-dispersed. *S. mellifera* produces papery, indehiscent pods (Palgrave, 1983; van Wyk & van Wyk, 1997), whereas *R. trichotomum* has winged seeds within a long, flattened capsule that appears to burst open several weeks after maturing (*pers. obs.*). Both of these species are thought to be wind-dispersed. Thus, we only expected to find seeds from the *Vachellia* genus intact within dung, specifically *V. erioloba*, *V. haematoxylon*, and *V. hebeclada*. These species produce indehiscent pods, a trait that often indicates reliance on mammalian herbivores for endozoochorous seed dispersal (Lamprey et al., 1974; M. F. Miller, 1995; M. F. Miller & Coe, 1993; Pellew & Southgate, 1984).(Lamprey et al., 1974; M. F. Miller, 1995; M. F. Miller & Coe, 1993; Pellew & Southgate, 1984). However, a seed’s resilience to consumption is also influenced by the thickness of its testa (Coe & Coe, 1987).

Given that *V. haematoxylon* has been identified as an abundant and key resource for black rhinos (Shaw, 2011), we assume that a large number of its pods are consumed, so would expect seeds to be present within dung boluses. Upon inspection, however, we observed that *V. haematoxylon* seeds are considerably smaller than *V. erioloba* seeds, with an estimated 70% of seeds in most pods appearing underdeveloped and potentially inviable. Furthermore, they are enclosed in more brittle pods than *V. erioloba* seeds. We presume that, while *V. haematoxylon* pods are consumed by rhinos, their seeds cannot withstand gut passage and are digested. In Tswalu, black rhinos have been observed feeding on *V. hebeclada* shrubs (*pers. obs.*). *V. hebeclada* seeds are more comparable in size to *V. erioloba* seeds and are protected by harder pods than *V. haematoxylon* (though thinner than *V. erioloba* pods). It is likely that *V. hebeclada* seeds are capable of surviving endozoochory; however, the low densities of this shrub in Tswalu may explain the absence of its seeds in our sampled boluses. Further research is needed on these two species, however , this study has become a case study of *V. erioloba*.

Surprisingly, during later harvests of black rhino dung, around January of the following year, stones from *Grewia flava* drupes were found in low densities within boluses. These were collected for separate germination and viability assessments (beyond the scope of this study). Additionally, while no seeds were recovered in dung, black rhinos were observed feeding on the gemsbok cucumber (*Acanthosicyos naudinianus*; Sond. C. Jeffrey; Cucurbitaceae), a herbaceous perennial with extremely hard seeds, potentially making it a strong candidate for herbivore-mediated dispersal and germination.

Details of HCl treatment:

Generally, sulfuric acid (H₂SO₄) and sodium hypochlorite (NaClO) are used in standard acid scarification protocols for germinating Acacia and other species (Daffalla et al., 2022; Kheloufi et al., 2019; Oyebamiji et al., 2023; Sorensen & Jusaitis, 1995; Yousif et al., 2020). We selected hydrochloric acid (HCl) for this study because it is a primary component of gastric acid, making it widely used to simulate digestion in hindgut fermenters (Alhuthayli et al., 2024; Longland et al., 2021; Moore-Colyer et al., 2020).

We sourced an average gut passage time for Desert Black Rhinos (50-60 hrs) based on comparative data from field and zoo studies (see Dierenfield, 1993). This was crucial to our study, as exposure time in the gut should significantly influence the effect on seed germinability (Clauss et al., 2005). Additionally, we sourced values for the pH levels of each section of the gastrointestinal tract. This data was measured in three black rhino individuals that were culled during legal wildlife control procedures (Clemens & Maloiy, 1982). Together, these figures informed our protocol to replicate the acid exposure that seeds would undergo in the black rhino gut: This data was measured in three black rhino individuals that were culled during legal wildlife control procedures (Clemens & Maloiy, 1982). Together, these figures informed our protocol to replicate the acid exposure that seeds would undergo in the black rhino gut:

In black rhino, most regions of the alimentary canal maintain a near-neutral pH, except for the cranial and caudal stomachs, which are notably acidic (Clemens & Maloiy, 1982). Thus, we expected acid scarification would to primarily occur during digestion in these regions. To replicate this process, seeds were soaked at room temperature within HCl solutions diluted to match these pH levels. The average pH of the cranial stomach is 4.48, equivalent to a 3.31 × 10⁻⁵ M HCl solution, while the caudal stomach averages a pH of 2.01, or 9.77 × 10⁻³ M HCl (Clemens & Maloiy, 1982).

Since the gut passage time in black rhinos averagely ranges from 50 to 60 hours (Dierenfield, 1993) but retention times for specific regions of the digestive tract are unknown, we conservatively simulated gut passage by soaking seeds for 20 hours in a 0.000033 M HCl solution, followed by another 20 hours in a 0.00977 M HCl solution. Seeds were not rinsed between or after these treatments to allow for any effects of residual acid, simulating conditions post-gut passage.

Details of GA treatment:

After treatment with HCl, seeds were submerged in a solution of 50 mg/L gibberellic acid (GA3; Kimix, BioReagent, suitable for plant cell culture, purity ≥ 90%) at room temperature and for 8 hours. Gibberellins, including GA3, are endogenous plant growth regulators (Tyagi et al., 2021) that stimulate seed germination by interacting with external factors such as light, temperature and water to promote transitional growth processes like meristem root and shoot elongation (Othman & Leskovar, 2022). Typically, seeds are soaked in GA₃ for 12-24 hours (Kashid et al., 2023; Praticia S et al., 2024; Yadav et al., 2023); however, they risk drowning after 48 hours of submersion. Since the seeds had already undergone a total of 40 hours of soaking in hydrochloric acid, we limited the GA₃ treatment to 8 hours, as in Kumar et al. (2024), albeit at a lower concentration.

Since the acids used in the GA treatment should break the physical and physiological dormancy of the seeds, respectively, this group was expected to yield the maximum genetic potential for germination in each seed. Thus, the GA and Control treatments were designed to establish minimum and maximum germination benchmarks for *V. erioloba* seeds, providing a comparative framework for the Dung and HCl treatment groups.

*2.5 Data analysis*

*Experiments 3 and 4: Monitoring early seedling development and clipping, to simulate black rhino herbivory*

Details of distribution identification using fitdistrplus:

The best fitting distribution for our models was decided upon by means of a deductive process. Tests for normality and homoscedasticity were carried out using residual diagnostic plots. The data had a normal fit but portrayed some unequal variance; thus, a semi-parametric approach was required. Candidate distributions were identified and tested by means of the *fitdistrplus* package (version 1.2-1; Delignette-Muller & Dutang, 2015). First, the *descdist* function was utilised to print unbiased estimations of skewness and Pearsons’s kurtosis values (Sokal & Rohlf, 2013). The growth data was found to be moderately right skewed (leptokurtic), whereas the recovery data was highly right skewed.

This function also generates a skewness-kurtosis plot such as the one proposed by Cullen and Frey (1999). On this plot, values for common distributions are displayed, which guided our choice of candidate distributions. They were the normal, lognormal, logistic, exponential and gamma distributions. For manual assessment, these were individually fitted to the predictor variable using a list of objects of class "fitdist". The function *plotdist* was then utilised to display all distributions on four classical Goodness-of-fit plots (Cullen & Frey, 1999): a cdf plot (function *cdfcomp*), a density plot (function *denscomp*), a density Q-Q plot (function *qqcomp*), or a P-P plot (function *ppcomp*).Once the best distribution was visually estimated, the function *gofstat* was utilised to print Goodness-of-fit statistics and Goodness-of-fit criteria. The former consisted of Kolmogorov-Smirnov, Cramer-von Mises, and Anderson-Darling statistics (D’Agostino & Stephens, 1986). The latter included both the Akaike and Bayesian Information Criterion.

**Table S1A.** Candidate models list (Experiment 3).

| **Candidate** | **Model Structure** |
| --- | --- |
| 1 | glmer(stem_area ~ 1 + (1\|ID), family = Gamma(link = "log"), data = precut_nz_clean) |
| 2 | glmer(stem_area ~ seedling_age_days + treatment + SM + (1\|ID), family = Gamma(link = "log"), data = precut_nz_clean, glmerControl(optimizer = "bobyqa", optCtrl = list(maxfun = 100000))) |
| 3 | glmer(stem_area ~ seedling_age_days + treatment + SM + T_Max + (1\|ID), family = Gamma(link = "log"), data = precut_nz_clean, glmerControl(optimizer = "bobyqa", optCtrl = list(maxfun = 100000))) |
| 4 | glmer(stem_area ~ seedling_age_days + treatment + SM + T_Max + SM*T_Max + (1\|ID), family = Gamma(link = "log"), data = precut_nz_clean, glmerControl(optimizer = "bobyqa", optCtrl = list(maxfun = 100000))) |
| 5 | glmer(stem_area ~ seedling_age_days + treatment + SM + pot_density + (1\|ID), family = Gamma(link = "log"), data = precut_nz_clean, glmerControl(optimizer = "bobyqa", optCtrl = list(maxfun = 100000))) |
| 6 | glmer(stem_area ~ seedling_age_days + treatment + SM + SM*pot_density + (1\|ID), family = Gamma(link = "log"), data = precut_nz_clean, glmerControl(optimizer = "bobyqa", optCtrl = list(maxfun = 100000))) |
| 7 | glmer(stem_area ~ seedling_age_days + treatment + SM + T_Max + pot_density + (1\|ID), family = Gamma(link = "log"), data = precut_nz_clean, glmerControl(optimizer = "bobyqa", optCtrl = list(maxfun = 100000))) |
| 8 | glmer(stem_area ~ seedling_age_days + treatment + SM + T_Max + SM*T_Max + pot_density + (1\|ID), family = Gamma(link = "log"), data = precut_nz_clean, glmerControl(optimizer = "bobyqa", optCtrl = list(maxfun = 100000))) |
| 9 – selected model | glmer(stem_area ~ seedling_age_days + treatment + SM + SM*pot_density + pot_density + T_Max + (1\|ID), family = Gamma(link = "log"), data = precut_nz_clean, glmerControl(optimizer = "bobyqa", optCtrl = list(maxfun = 100000))) |
| 10 | glmer(stem_area ~ seedling_age_days + treatment + substrate + pot_density + SM + T_Max + (1\|ID), family = Gamma(link = "log"), data = precut_nz_clean, glmerControl(optimizer = "bobyqa", optCtrl = list(maxfun = 100000))) |
| 11 | glmer(stem_area ~ seedling_age_days + treatment + substrate + pot_density + SM*pot_density + SM + T_Max + (1\|ID), family = Gamma(link = "log"), data = precut_nz_clean, glmerControl(optimizer = "bobyqa", optCtrl = list(maxfun = 100000))) |

**Table S1B.** Candidate models AICc output (Experiment 3).

| **Model** | **K** | **AICc** | **Delta AICc** | **AICc Wt** | **Cum Wt** | **LL** |
| --- | --- | --- | --- | --- | --- | --- |
| **mod 9** | 11 | 895.3696 | 0.0000 | 0.4775 | 0.4775 | - 435.9348 |
| **mod 6** | 10 | 896.7552 | 1.3856 | 0.2388 | 0.7163 | - 437.7561 |
| **mod 11** | 12 | 897.5985 | 2.2289 | 0.1567 | 0.8730 | - 435.9078 |
| **mod 3** | 9 | 899.9089 | 4.5393 | 0.0493 | 0.9224 | - 440.4488 |
| **mod 7** | 10 | 901.7292 | 6.3596 | 0.0199 | 0.9422 | - 440.2431 |
| **mod 4** | 10 | 901.8900 | 6.5204 | 0.0183 | 0.9605 | - 440.3235 |
| **mod 2** | 8 | 902.0103 | 6.6407 | 0.0173 | 0.9778 | - 442.6029 |
| **mod 5** | 9 | 903.5956 | 8.2260 | 0.0078 | 0.9856 | - 442.2922 |
| **mod 8** | 11 | 903.6470 | 8.2774 | 0.0076 | 0.9932 | - 440.0735 |
| **mod 10** | 11 | 903.8794 | 8.5098 | 0.0068 | 1.0000 | - 440.1897 |
| **mod 1** | 3 | 947.9313 | 52.5617 | 0.0000 | 1.0000 | - 470.9004 |

**Table S2A.** Candidate models list (Experiment 4).

| **Candidate** | **Model Structure** |
| --- | --- |
| 1 | glmer(stem_area ~ 1 + (1\|ID), family = Gamma(link = "log"), data = postcut_2_clean) |
| 2 | glmer(stem_area ~ T_Max + substrate + seedling_age_days + (1\|ID), family = Gamma(link = "log"), data = postcut_2_clean, glmerControl(optimizer = "bobyqa", optCtrl = list(maxfun = 100000))) |
| 3 | glmer(stem_area ~ T_Max + substrate + seedling_age_days + substrate*T_Max + (1\|ID), family = Gamma(link = "log"), data = postcut_2_clean, glmerControl(optimizer = "bobyqa", optCtrl = list(maxfun = 100000))) |
| 4 | glmer(stem_area ~ T_Max + substrate + seedling_age_days + SM_avg + (1\|ID), family = Gamma(link = "log"), data = postcut_2_clean, glmerControl(optimizer = "bobyqa", optCtrl = list(maxfun = 100000))) |
| 5 – selected model | glmer(stem_area ~ T_Max + substrate + seedling_age_days + SM_avg + substrate*T_Max + (1\|ID), family = Gamma(link = "log"), data = postcut_2_clean, glmerControl(optimizer = "bobyqa", optCtrl = list(maxfun = 100000))) |
| 6 | glmer(stem_area ~ seedling_age_days + treatment + substrate + pot_density + SM_avg + T_Max + (1\|ID), family = Gamma(link = "log"), data = postcut_2_clean, glmerControl(optimizer = "bobyqa", optCtrl = list(maxfun = 100000))) |
| 7 | glmer(stem_area ~ seedling_age_days + treatment + substrate + pot_density + SM_avg + T_Max + substrate*T_Max + (1\|ID), family = Gamma(link = "log"), data = postcut_2_clean, glmerControl(optimizer = "bobyqa", optCtrl = list(maxfun = 100000))) |

**Table S2B.** Candidate models AICc output (Experiment 4).

| **Model** | **K** | **AICc** | **Delta AICc** | **AICc Wt** | **Cum Wt** | **LL** |
| --- | --- | --- | --- | --- | --- | --- |
| **mod 5** | 8 | 1141.583 | 0.0000 | 0.5566 | 0.5566 | - 562.5083 |
| **mod 4** | 7 | 1142.347 | 0.7633 | 0.3800 | 0.9366 | - 563.9538 |
| **mod 7** | 12 | 1147.004 | 5.4201 | 0.0370 | 0.9736 | - 560.8778 |
| **mod 6** | 11 | 1147.681 | 6.0981 | 0.0264 | 1.0000 | - 562.3149 |
| **mod 3** | 7 | 1319.242 | 177.6583 | 0.0000 | 1.0000 | - 652.4330 |
| **mod 2** | 6 | 1322.056 | 180.4726 | 0.0000 | 1.0000 | - 654.8876 |
| **mod 1** | 3 | 1847.001 | 705.4171 | 0.0000 | 1.0000 | - 920.4687 |

**3. Results**

*3.1 Germination assays*

*Experiment 1: Disentangling the effects of gut passage on germination*

The cumulative hazard curve H(t) represents the accrued ‘risk’ of germination over time, as reflected in the calculated hazard ratio values. The cumulative hazard curves (Figure S1A) show that Dung seeds and those treated with gibberellic acid (GA) and hydrochloric acid (HCl) accrued a significantly higher risk of germination over time compared to the control group. The hazard ratios (Figure S1B) further highlight these trends, with GA-treated seeds showing the highest relative germination likelihood (HR = 1.9, 95 % CI 1.5−2.3, p<0.001), followed closely by dung (HR = 1.7, 95 % CI 1.4−2.11, p<0.001) and HCl (HR = 1.7, 95 % CI 1.4−2.1, p<0.001). The comparable performance of the dung treated seeds to even the GA treatment group, included as a maximum germination benchmark, underscores the efficacy of gut passage in enhancing germination, with significantly higher germination probabilities compared to untreated seeds in the control group. This supports the hypothesis that endozoochory by black rhinos can aid *V. erioloba* in overcoming dormancy barriers.

**Table S3.** Output from the pairwise comparison of the log-rank test, which assessed the survival probabilities of seeds across all treatment groups over time. Treatment groups consisted of seeds collected from dung, seeds collected from pods and either left untreated as a control, treated with hydrochloric acid (HCl) or additionally treated with gibberellic acid (GA).

| **Treatment** | **Control** | **Dung** | **Gibberellic Acid (GA)** |
| --- | --- | --- | --- |
| **Dung** | <0.0001*** | - | - |
| **Gibberellic Acid (GA)** | <0.0001*** | 0.44 | - |
| **Hydrochloric Acid (HCl)** | <0.0001*** | 0.87 | 0.44 |

**Table S4.** P-values (**p**) and Wald statistics (**z**) from the Cox proportional hazards test, which assessed the germination probabilities of seeds across all treatment groups relative to the Control, while accounting for the risk of germination occurring that has accrued over time. Treatment groups consisted of seeds collected from dung, seeds collected from pods and either left untreated as a control, treated with hydrochloric acid (HCl) or additionally treated with gibberellic acid (GA).

| **Treatment** | **Output** | **Dung** | **Hydrochloric Acid (HCl)** | **Gibberellic Acid (GA)** |
| --- | --- | --- | --- | --- |
| **Control** | p | <0.0001*** | <0.0001*** | <0.0001*** |
|  | z | 4.859 | 4.735 | 5.712 |


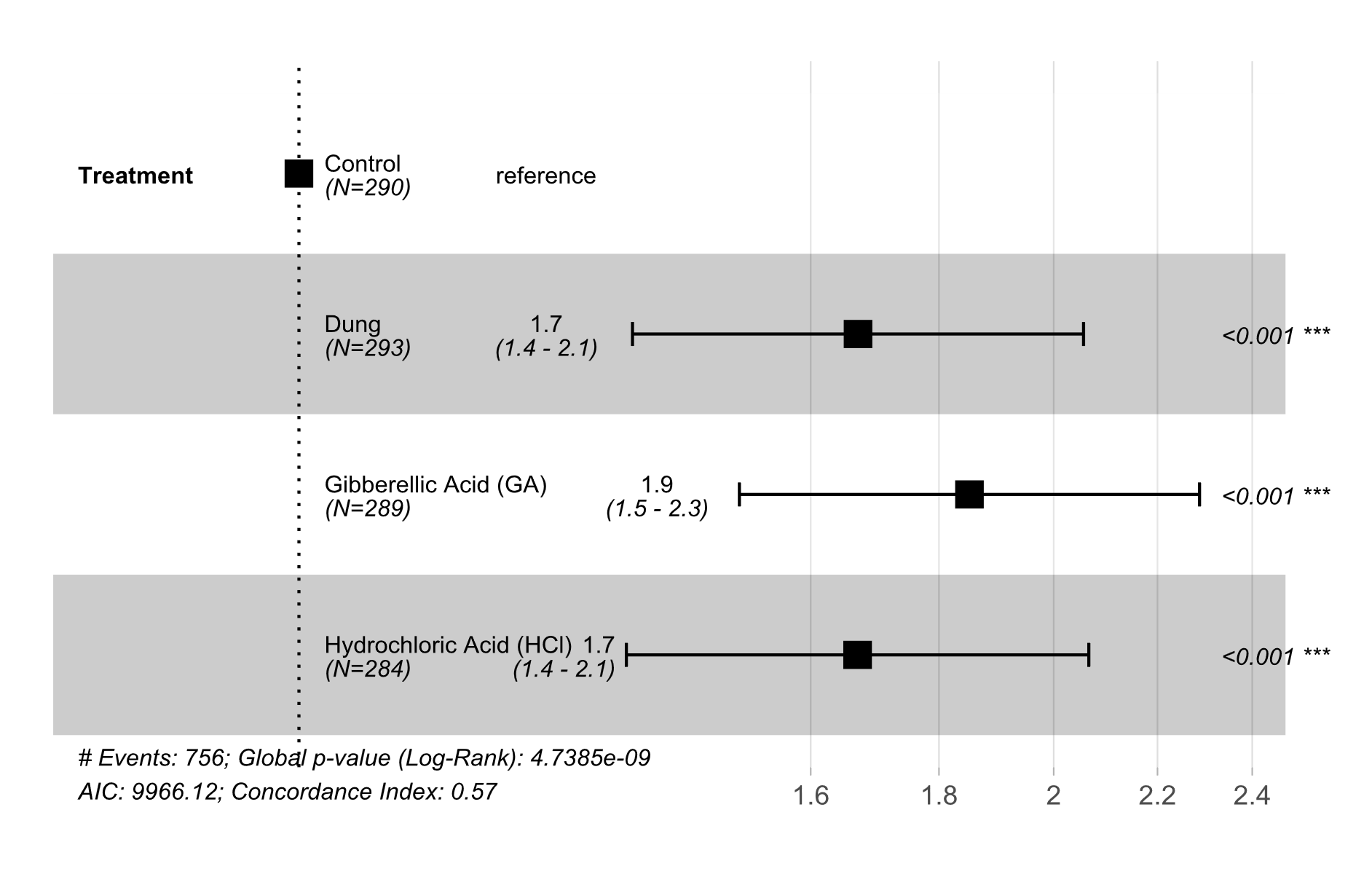

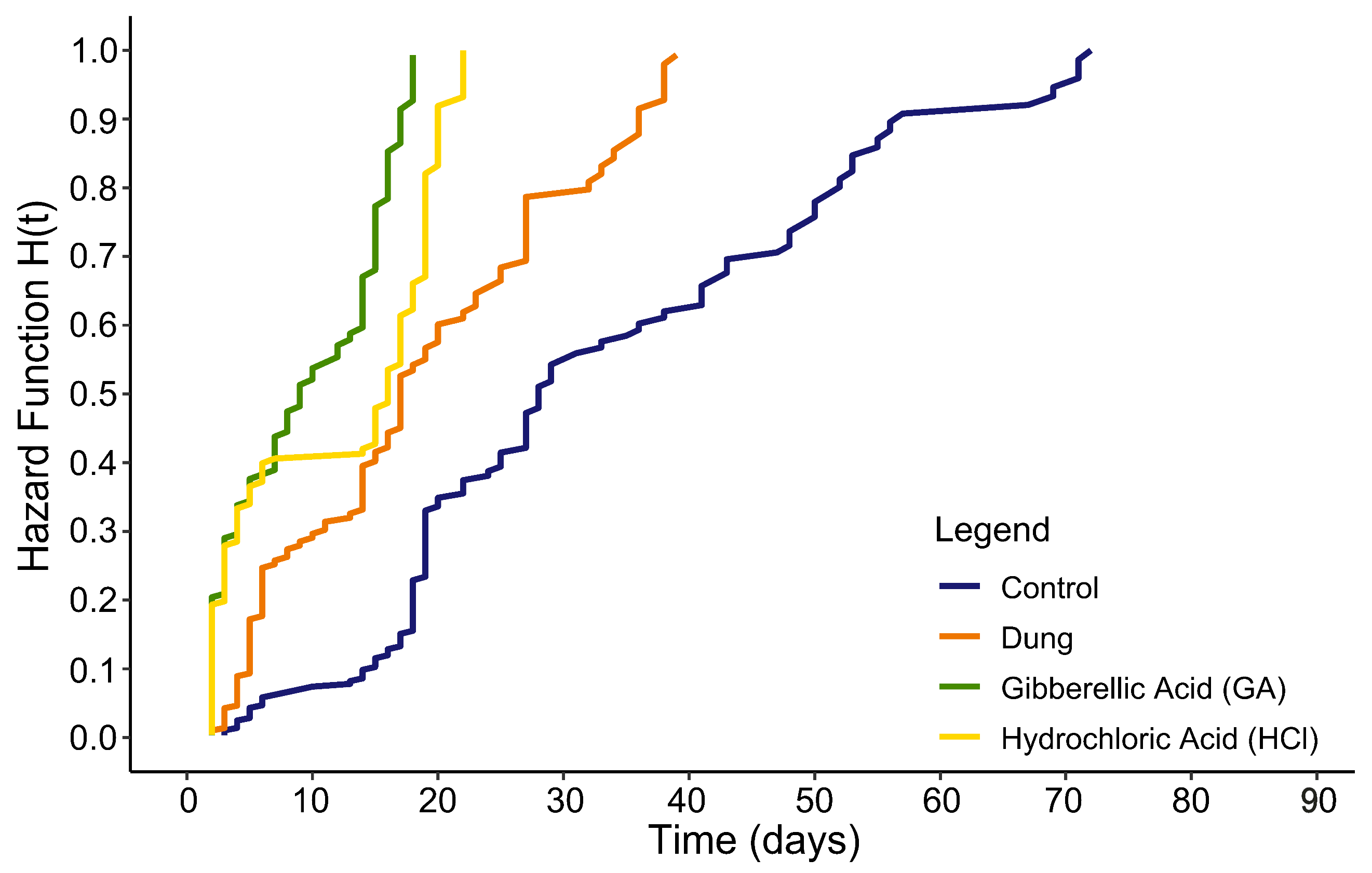


A

B

**Figure S1.** Hazard step curves (**A**) and hazard ratios (**B**) for control, dung, hydrochloric acid, and gibberellic acid-treated seed samples over a period of 86 days (~11 weeks).

*Experiment 2: Assessing the impact of field exposure on seed germinability*

Seeds collected from dung in April exhibited the highest germination likelihood (HR = 1.59, 95 % CI 1.31−1.91, p<0.001), significantly outperforming all other treatment groups. In contrast, seeds collected from dung in July had a much lower hazard ratio, even lower than the control groups (HR = 0.31, 95 % CI 0.23−0.41, p<0.001; Figure S2A), suggesting that prolonged exposure to environmental factors negatively impacts seed viability. These results underscore the importance of early consumption, dispersal, and germination facilitation by herbivore mutualists such as black rhinos.

**Table S5.** Output from the pairwise comparison of the log-rank test, which assessed the survival probabilities of seeds across all treatment groups over time. Treatment groups consisted of seeds collected from dung in April and July. We included two sets of control seeds, both collected from mature, fallen pods in April.

| **Treatment** | **Control 1** | **Control 2** | **Dung April** |
| --- | --- | --- | --- |
| **Control 2** | 0.069 | - | - |
| **Dung April** | <0.0001*** | <0.0001*** | - |
| **Dung July** | <0.0001*** | <0.0001*** | <0.0001*** |

**Table S6.** P-values (**p**) and Wald statistics (**z**) from the Cox proportional hazards test, which assessed the germination probabilities of seeds across all treatment groups relative to Control 1, while accounting for the risk of germination occurring that has accrued over time. Treatment groups consisted of seeds collected from dung in April and July. We included two sets of control seeds, both collected from mature, fallen pods in April.

| **Treatment** | **Output** | **Control 2** | **Dung April** | **Dung July** |
| --- | --- | --- | --- | --- |
| **Control 1** | p | 0.0837 | <0.0001*** | <0.0001*** |
|  | z | -1.730 | 4.797 | -8.066 |


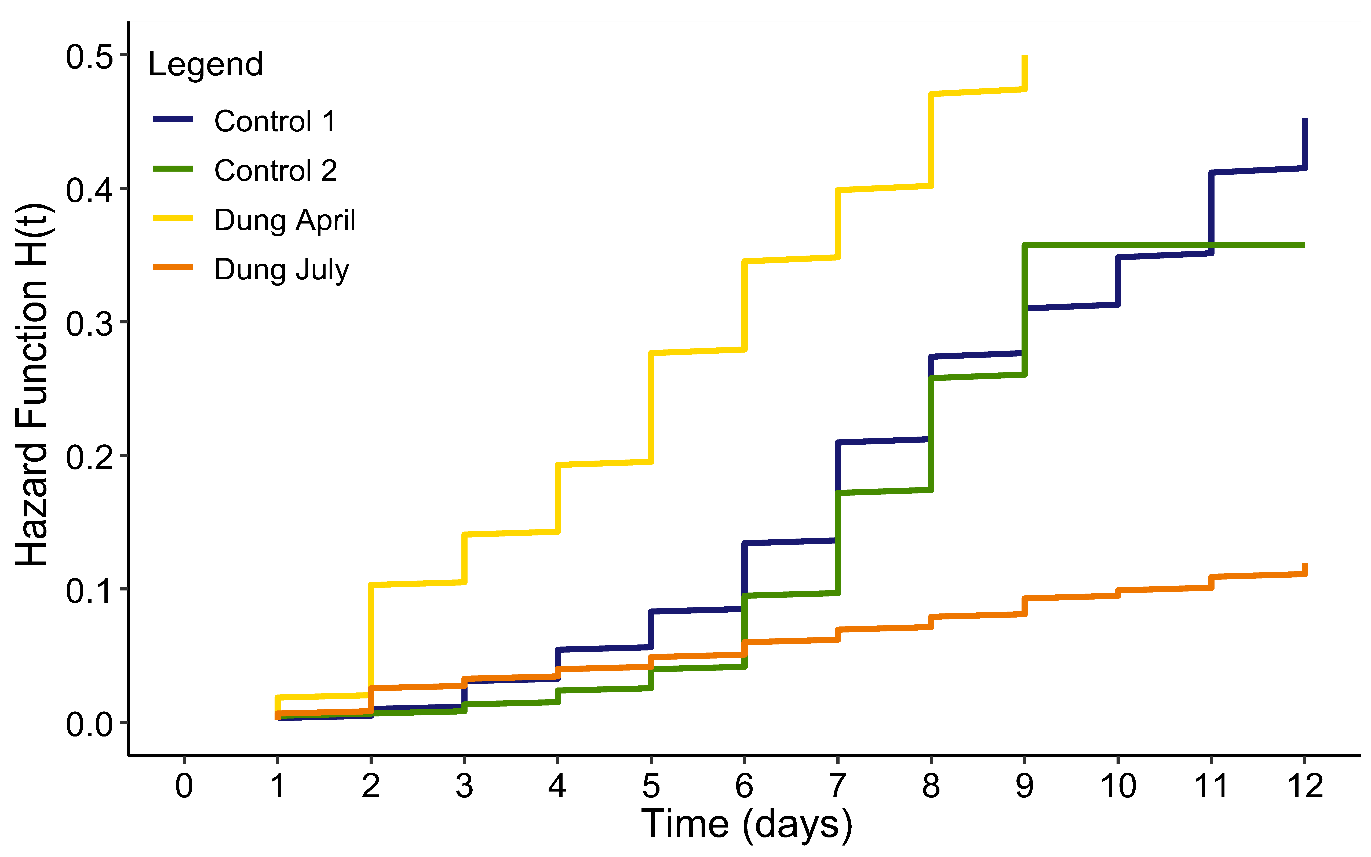

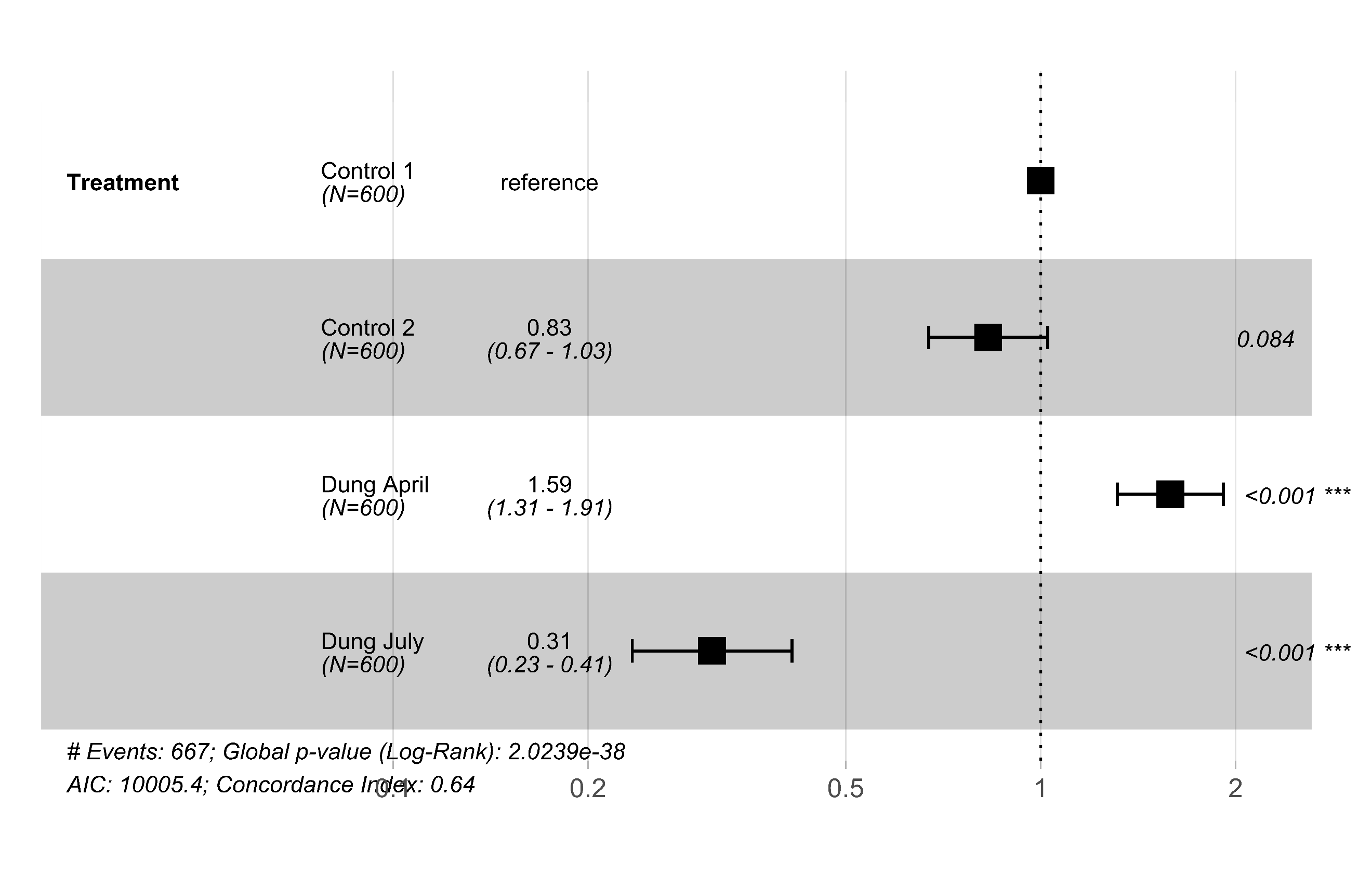


A

B

**Figure S2.** Hazard step curves (**A**) and hazard ratios (**B**) for seeds collected from dung in April and July, as well as two control groups, over a period of 12 days.

*3.2 Testing early seedling growth and resilience*

*Experiment 3: Monitoring early seedling development*

**Table S7.** Generalized Linear Mixed-Effects Model (GLMM) output for early seedling growth (from ~60 to ~153 days old).

| stem_area ~ seedling_age_days + treatment + SM + pot_density  + SM : pot_density + T_Max + (1 \| ID) | | | | | |
| --- | --- | --- | --- | --- | --- |
| Coefficients |  | Estimate | Std error | t value | p |
| Intercept (treatment: Control) |  | 2.0795248 | 0.80982073 | 2.567883 | 0.01023217 |
| seedling_age_days |  | 0.2226719 | 0.05846551 | 3.808602 | 0.00013975 |
| treatment: Dung |  | 1.1118104 | 0.66256944 | 1.678028 | 0.09334155 |
| treatment: GA |  | 2.1087204 | 0.59741154 | 3.529762 | 0.00041593 |
| Treatment: HCl |  | 1.9816366 | 0.58909174 | 3.363884 | 0.00076854 |
| soil_moisture (SM) |  | -13.7633909 | 2.87788559 | -4.782466 | 0.00000173 |
| pot_density |  | -0.7659855 | 0.34086151 | -2.247204 | 0.02462697 |
| T_Max |  | -0.1030494 | 0.05397057 | -1.909363 | 0.05621529 |
| SM : pot_density |  | 4.5244834 | 1.53135899 | 2.954554 | 0.00313121 |

*Experiment 4: Clipping, to simulate black rhino herbivory*

**Table S8.** Generalized Linear Mixed-Effects Model (GLMM) output for the clipping experiment. Seedlings were clipped at ~102 to ~153 days old.

| stem_area ~ T_Max + substrate + seedling_age_days + SM + substrate * T_Max + (1 \| ID) | | | | | |
| --- | --- | --- | --- | --- | --- |
| Coefficients |  | Estimate | Std error | t value | p |
| Intercept (substrate: dung) |  | 1.591044 | 0.191653 | 8.302 | 0.00000000 |
| T_Max |  | 0.009745 | 0.040601 | 0.240 | 0.81030759 |
| substrate: sand |  | 0.743730 | 0.185460 | 4.010 | 0.00006067 |
| seedling_age_days |  | 0.082204 | 0.024466 | 3.360 | 0.00077952 |
| Soil_moisture (SM) |  | 1.108474 | 0.817277 | 1.356 | 0.17500330 |
| T_Max:substrate(sand) |  | -0.073999 | 0.043408 | -1.705 | 0.08824686 |

**4. Discussion**

*4.1 Black rhino increase germination rates and germination duration*

The lighter colour and softer texture of dung seeds, compared to pod seeds, were initial signs of seed coat weakening. In Fabaceae, the dark pigmentation of the seed coat is caused by the presence of phenolics, that maintain physical dormancy by contributing to water-impermeability (Smýkal et al., 2014; Tran & Cavanagh, 1984). Most prominently, dung seeds exhibited linear fissures in their centre and an open pleurogram. This U-shaped groove on both seed faces is considered a point of structural weakness, that acts as a one-way, permeable valve and may play a role in regulating dormancy breakage and germination during seed maturation (Rodrigues-Junior et al., 2021). These observations suggested that gut passage facilitates water imbibition and triggers dormancy breakage, as shown by Tran and Cavanagh (1984) and Hoffman et al. (1989). This hypothesis was tested in the first germination trial.

**Additional References**

Alhuthayli, H. F., A. Mohammed, M., Al-Kharashi, N. A., Al-Jasass, F. M., A. Yagoub, A. E., & A. Osman, M. (2024). Impact of simulated *in vitro* gastrointestinal digestion on phenolic compounds and the antioxidant potential of olive pomace. *Italian Journal of Food Science*, 36(3), 276–290. <https://doi.org/10.15586/ijfs.v36i3.2665>

Cullen, A., & Frey, H. C. (1999). *Probabilistic techniques in exposure assessment: A handbook for dealing with variability and uncertainty in models and inputs.* New York, NY: Plenum Press.

Daffalla, H. M., Ali, K. S., Osman, M. G., & Yahiya, Y. O. (2022). Rapid germination and development of *Acacia sieberiana* DC *in vitro*. *Notulae Scienta Biologicae*, 14(2), 1–14. <https://doi.org/10.15835/nsb14211176>

D’Agostino, R., & Stephens, M. A. (1986). *Goodness-of-fit techniques*. New York, NY: CRC Press.

Kashid, U. V., Nelson Navamaniraj, K., Umarani, R., Umesh Kanna, S., & Masilamani, P. (2023). Reviving the resilient: Exploring pre-dormancy breaking treatments for *Acacia nilotica* (L.). *International Journal of Environment and Climate Change,* 13(10), 2063–2070. <https://doi.org/10.9734/ijecc/2023/v13i102867>

Kheloufi, A., Boukhatem, Z. F., Mansouri, L. M., & Djelilate, M. (2019). Maximizing seed germination in five species of the genus *Acacia* (Fabaceae). *Reforesta*, 7(1), 15–23. <http://dx.doi.org/10.21750/REFOR.7.02.64>

Longland, A. C., Barfoot, C., & Harris, P. A. (2021). The degradation of four different horse feeds *in vitro* previously evaluated in ponies *in vivo*. *Animal Feed Science and Technology*, 279(1), 115-121. <https://doi.org/10.1016/j.anifeedsci.2021.115021>

Moore-Colyer, M., Tuthill, P., Bannister, I., & Daniels, S. (2020). Growth rates of thoroughbred foals and i*n vitro* gut health parameters when fed a cereal or an all-fiber creep feed. *Journal of Equine Veterinary Science*, 93(1), 103-191. <https://doi.org/10.1016/j.jevs.2020.103191>

Othman, Y. A., & Leskovar, D. I. (2022). Foliar application of gibberellic acid improves yield and head phenolic compounds in globe artichoke. *Scientia Horticulturae*, 301(1), 1–9. <https://doi.org/10.1016/j.scienta.2022.111115>

Oyebamiji, N. A., Ojekunle, O. O., Opanike, O. O., & Yisau, J. A. (2023). Effects of pre-sowing techniques on selected seeds of savanna agroforestry tree species. *Journal of the Cameroon Academy of Sciences*, 19(1), 31–41. <https://doi.org/10.4314/jcas.v19i1.3>

Praticia, S. P. J., Kesavan, R. K. S., Kanchana, M. (2024). Unlocking growth potential: Enhancing salt-stressed seed germination and seedling growth with Ga3 priming in *Acacia auriculiformis* A. cunn. ex Benth., *Delonix regia* (Bojer ex Hook.) Raf. and *Cassia fistula L. Current Agriculture Research Journal*, 11(3), 1004–1012. <https://doi.org/10.12944/CARJ.11.3.28>

Rodrigues-Junior, A. G., Baskin, C. C., Baskin, J. M., & De-Paula, O. C. (2021). The pleurogram, an under-investigated functional trait in seeds. *Annals of Botany*, *127*(2), 167–174. <https://doi.org/10.1093/aob/mcaa161>

Smýkal, P., Vernoud, V., Blair, M. W., Soukup, A., & Thompson, R. D. (2014). The role of the testa during development and in establishment of dormancy of the legume seed. *Frontiers in Plant Science*, *5*. <https://doi.org/10.3389/fpls.2014.00351>

Sokal, R., & Rohlf, F. (2013). *Biometry: The Principles and Practice of Statistics in Biological Research* (4th ed.). New York, NY: W.H. Freeman and Company.

Sorensen, B., & Jusaitis, M. (1995). Seed germination of endangered south Australian plants. *Combined Proceedings lnternational Plant Propagators' Society*, 45, 86–92.

Tyagi, K., Maoz, I., Kochanek, B., Sela, N., Lerno, L., Ebeler, S. E., & Lichter, A. (2021). Cytokinin but not gibberellin application had major impact on the phenylpropanoid pathway in grape. *Horticulture Research*, 8(1), 1-51. <https://doi.org/10.1038/s41438-021-00488-0>

Yadav, A., Bohra, N. K., & Giri, V. (2023). Study the effect of plant growth regulators on seed germination of various tree species of Rajasthan. *Asian Journal of Basic Science & Research*, 5(3), 94–100. <https://doi.org/10.38177/AJBSR.2023.5308>

Yousif, M. A. I., Wang, Y. R., & Dali, C. (2020). Seed dormancy overcoming and seed coat structure change in *Leucaena leucocephala* and *Acacia nilotica*. *Forest Science and Technology*, 16(1), 18–25. https://doi.org/10.1080/21580103.2019.1700832
